# Consensus recommendations on perioperative mechanical ventilation strategies: an evidence-based guideline by the Brazilian Society of Anesthesiology

**DOI:** 10.1016/j.bjane.2026.844814

**Published:** 2026-08-18

**Authors:** João Manoel Silva-Jr, Talison Silas Pereira, Luiz Fernando dos Reis Falcão, Ricardo Esper Treml, Alexandre Marini Isola, Alexandre Goulart Pustilnik, Anacleto Gabriel de Alcântara, André Prato Schmidt, Antonio Carlos Aguiar Brandão, Arthur Caus de Morais, Bruna Silveira Ferreira Klauck, Bruno Campostrini Sily, Bruno Francisco de Freitas Tonelotto, Carlos Gustavo dos Santos Silva, Carmen Sílvia Valente Barbas, Claudia Marquez Simões, Daniel da Escóssia Melo Sousa, Fábio Barlem Hohmann, Fábio Luis Ferrari Regatieri, Germano Pinheiro Medeiros, Gutenberg Diniz Borborema, Maisa Ribeiro Araújo, Marcelo Ramalho Fernandes, Marcelo Tabary de Oliveira Carlucci, Matheus Faccini Vane, Neymar Elias de Oliveira, Plínio da Cunha Leal, Roberta Figueiredo Vieira, Rodrigo Leal Alves, Rodrigo Pereira Diaz André, Saullo Queiroz Silveira, Victor Sampaio de Almeida, Vinícius Caldeira Quintão, Leandro Gobbo Braz, Luiz Marcelo Sá Malbouisson

**Affiliations:** a Universidade de São Paulo (FMRP-USP), Faculdade de Medicina de Ribeirão Preto, Departamento de Cirurgia e Anatomia, Divisão de Medicina Intensiva, Ribeirão Preto, SP, Brazil; b Faculdade de Medicina da Universidade de São Paulo (FMUSP), Programa de Pós-Graduação em Anestesiologia, Ciências Cirúrgicas e Medicina Perioperatória, São Paulo, SP, Brazil; c Hospital do Servidor Público Estadual de São Paulo (HSPE-IAMSPE), São Paulo, SP, Brazil; d Universidade Federal de São Paulo (UNIFESP), Disciplina de Anestesiologia, Dor e Terapia Intensiva, São Paulo, SP, Brazil; e Stanford University School of Medicine, Department of Anesthesiology, Perioperative and Pain Medicine, Stanford, CA, USA; f Imed Group, Departamento de Educação Continuada, São Paulo, SP, Brazil; g Hospital São Rafael, Salvador, BA, Brazil; h Rede D'Or São Luiz, Departamento de Anestesiologia, Brasília, DF, Brazil; i Universidade Federal do Rio Grande do Sul (UFRGS), Departamento de Farmacologia, Serviço de Anestesiologia, Porto Alegre, RS, Brazil; j Hospital de Clínicas de Porto Alegre (HCPA), Porto Alegre, RS, Brazil; k Hospital das Clínicas Samuel Libânio – Faculdade de Ciências Médicas de Pouso Alegre (FCMPA), Pouso Alegre, MG, Brazil; l Universidade Estadual Paulista (UNESP), Faculdade de Medicina de Botucatu, Departamento de Especialidades Cirúrgicas e Anestesiologia, Botucatu, SP, Brazil; m Hospital das Clínicas da Faculdade de Medicina da Universidade de São Paulo (HCFMUSP), Instituto da Criança e do Adolescente (ICr), São Paulo, SP, Brazil; n Hospital Meridional Cariacica, VITALLE Anestesia e Cuidados Perioperatórios, Cariacica, ES, Brazil; o Hospital Sírio-Libanês, Serviço Médico de Anestesia (SMA), São Paulo, SP, Brazil; p Instituto Dante Pazzanese de Cardiologia, Departamento de Cirurgia e Anestesia, São Paulo, SP, Brazil; q Universidade de São Paulo, INCOR, Pneumology, São Paulo, SP, Brazil; r Hospital Israelita Albert Einstein, Departamento de Cuidados Críticos, São Paulo, SP, Brazil; s Instituto Paulista de Assistência Respiratória (IPAR), Hospital São Camilo Pompéia, São Paulo, SP, Brazil; t Hospital Geral de Fortaleza, Fortaleza, CE, Brazil; u Hospital de Emergência e Trauma Senador Humberto Lucena, João Pessoa, PB, Brazil; v Universidade Federal de Goiás, Goiânia, GO, Brazil; w Hospital Israelita Albert Einstein, Goiânia Unit, Goiânia, GO, Brazil; x Serviço de Anestesiologia Cardiovascular, Hospital Pró-Cardíaco / Hospital Copa Star / Hospital Copa D’Or, Rio de Janeiro, RJ, Brazil; y A.C.Camargo Cancer Center, Serviço de Anestesiologia, São Paulo, SP, Brazil; z Laboratório de Investigação Médica da Anestesiologia (LIM08), Faculdade de Medicina da Universidade de São Paulo (FMUSP), São Paulo, SP, Brazil; aa Faculdade de Ciências Médicas de São José dos Campos – Humanitas, São José dos Campos, SP, Brazil

**Keywords:** Anesthesia, GRADE, Mechanical ventilation, Perioperative care, Protective ventilation, Pulmonary complications

## Abstract

**Background:**

Postoperative Pulmonary Complications (PPCs) are a leading cause of morbidity, mortality, and prolonged hospitalization in surgical patients. Intraoperative mechanical ventilation plays a critical role in mitigating these risks, yet clinical practices remain heterogeneous and often not guided by evidence-based standards.

**Objective:**

To develop evidence-based recommendations on perioperative mechanical ventilation strategies for surgical patients, aiming to optimize respiratory care, reduce PPCs, and standardize practice in Brazil.

**Methods:**

This guideline was developed under the coordination of the Brazilian Society of Anesthesiology (SBA) by a panel of anesthesiologists and intensivists from multiple institutions. Seventeen clinical questions were structured in the PICO format (Population, Intervention, Comparison, Outcome). Systematic literature searches were conducted in PubMed, Embase, Cochrane Library, and Scopus up to July 2025. The strength of recommendations and certainty of evidence were assessed using the GRADE methodology. A modified Delphi process was applied to obtain agreement among collaborators, and consensus was discussed only when the predefined threshold of at least 80% agreement was not achieved. All recommendations were standardized in a uniform format.

**Results:**

Seventeen thematic chapters were developed, covering topics from respiratory physiology and the definition of PPCs to advanced intraoperative monitoring, special populations (obese, pediatric, thoracic and cardiac surgery), and patients with or without acute lung injury. Key recommendations include the routine use of lung-protective strategies low tidal volumes of 6–8 mL.kg⁻¹ PBW in patients without ARDS and 4–8 mL.kg⁻¹ PBW in patients with ARDS, Positive End-Expiratory Pressure [PEEP] titrated to physiology, driving pressure ΔP ≤ 15 cm H_2_O, and Plateau Pressure [Pplat] ≤ 30 cm H_2_O), individualized approaches in high-risk patients, avoidance of unnecessary high fraction of inspired oxygen (FiO_2_), and selective use of recruitment maneuvers. Standardized definitions of PPCs and structured monitoring were emphasized as essential to improving patient outcomes.

**Conclusion:**

These consensus-based recommendations provide clinicians with a practical, evidence-informed framework for perioperative mechanical ventilation. Adoption of protective strategies and standardized definitions is expected to improve respiratory outcomes and reduce variability in perioperative care.

## Introduction

Perioperative mechanical ventilation is a cornerstone of anesthetic and surgical management, directly influencing pulmonary outcomes and overall postoperative recovery.^1^ Postoperative Pulmonary Complications (PPCs) remain among the most frequent adverse events following surgery, and are strongly associated with increased morbidity, mortality, longer hospital stays, and higher healthcare costs.^2^ Their incidence can be mitigated through the adoption of protective ventilation strategies supported by evidence-based perioperative practices.^3^

## Respiratory physiology applied to perioperative mechanical ventilation

General anesthesia, neuromuscular blockade, mechanical ventilation, and surgical positioning induce profound changes in respiratory physiology.^4^^,^^5^ These include reductions in Functional Residual Capacity (FRC), airway closure in dependent lung regions, alveolar collapse (atelectasis), and reduced compliance of the respiratory system.^6^

The FRC, the volume of gas remaining in the lungs after passive expiration, typically decreases about approximately 0.4‒0.5 L during general anesthesia.^7^ This reduction approximates lung volume to closing capacity, predisposing small airways to collapse, particularly in dependent regions. The combination of controlled ventilation, neuromuscular blockade, and abdominal forces contributes to diaphragmatic dysfunction and cephalad displacement of the diaphragm.^8^ This mechanical shift further compresses dependent lung zones, exacerbating atelectasis and reducing FRC.^2^ As a result, ventilation becomes heterogeneous, with impaired compliance and increased elastance of the respiratory system. These changes lead to ventilation-perfusion mismatch, increased intrapulmonary shunt, and impaired oxygenation.^9^

## Perioperative clinical implications

**Figure 1:**
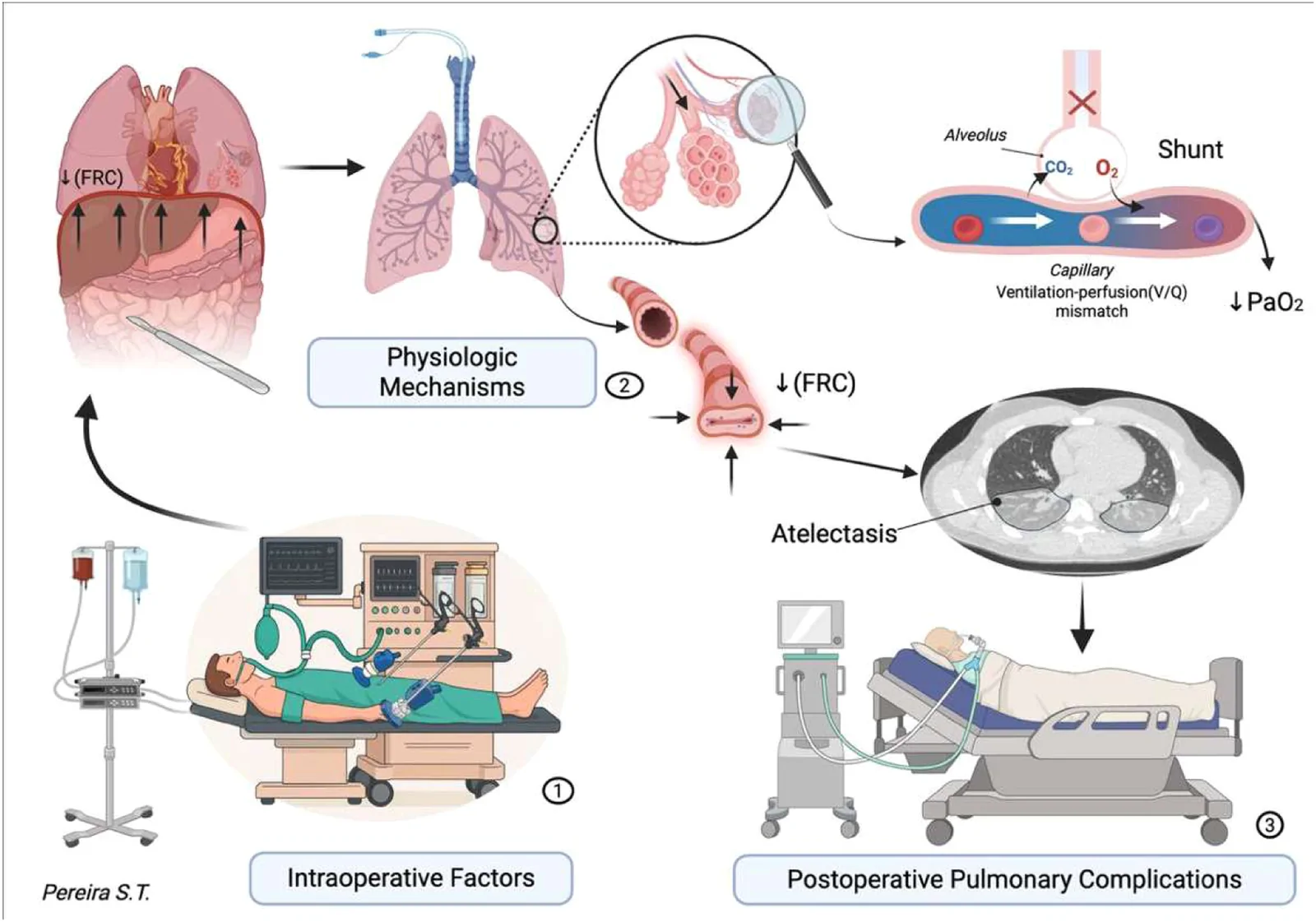
Schematic representation of respiratory physiology in the perioperative context. The central component depicts an anesthetized patient under mechanical ventilation during a surgical procedure, serving as the foundation for understanding subsequent physiological changes. Surrounding elements illustrate how general anesthesia, neuromuscular blockade, patient-related predisposing factors (such as obesity and aging), and surgical trauma contribute to diaphragmatic dysfunction, reduction in Functional Residual Capacity (FRC), alveolar collapse, and ventilation-perfusion (V̇/Q̇) mismatch. These changes are accompanied by impaired lung mechanics, including decreased static compliance. The graphic structure highlights the interplay between physiological mechanisms, anesthetic interventions, and postoperative risk, reinforcing the importance of preserving respiratory physiology as a cornerstone of lung-protective ventilation. Created in BioRender by Talison Silas Pereira (Pereira S.T.).

When FRC decreases toward closing capacity, the likelihood of airway closure increases.^6^ At the same time, elevated elastance and reduced compliance heighten susceptibility to Ventilator-Induced Lung Injury (VILI)^10^ (Fig. 1). General anesthesia further aggravates these effects by attenuating hypoxic pulmonary vasoconstriction and altering respiratory drive, thereby compromising gas exchange.^11^ A thorough understanding of these physiological mechanisms provides the basis for tailoring ventilatory strategies to individual patients and surgical contexts. By maintaining end-expiratory lung volume above closing capacity, reducing ventilation heterogeneity, and minimizing potentially injurious mechanical forces, clinicians can optimize respiratory care. This physiology-guided approach has been associated with a lower incidence of PPCs, shorter hospital stays, and reduced perioperative mortality.^1^

Although protective mechanical ventilation is well established in intensive care, particularly for patients with Acute Respiratory Distress Syndrome (ARDS),^12^ its systematic translation into intraoperative practice has been inconsistent. Unique aspects of the perioperative setting including surgical positioning, neuromuscular blockade, pneumoperitoneum during laparoscopy, and patient-specific conditions such as obesity or pre-existing lung disease require tailored ventilatory strategies.

In response to this need, the Brazilian Society of Anesthesiology (SBA) convened a panel of experts in anesthesiology and intensive care to develop a comprehensive, evidence-based guideline. The goal of this document is to provide clinicians with safe, protective, and individualized ventilation strategies for surgical patients, based on the best available scientific evidence and adapted to real-world perioperative practice.

This guideline is structured around 17 clinical questions developed in the PICO format (Population, Intervention, Comparison, Outcome). Recommendations are explicitly graded according to the GRADE system (Grading Recommendations, Assessment, Development and Evaluation). Each chapter provides a rationale supported by the literature and a summary table to guide clinical application. This guideline does not establish a minimum standard of practice or replace clinical judgment. It should be used as a tool to support decision-making and not as a rigid protocol.

## Methods

This guideline was developed under the coordination of the Brazilian Society of Anesthesiology (SBA), following a structured and transparent process based on the principles recommended by the GRADE Working Group and international standards for the development of clinical practice guidelines.^13^, ^14^, ^15^, ^16^ The project involved anesthesiologists, intensivists, experts in perioperative medicine, and methodologists from different institutions across Brazil.

### Panel composition and scope

This guideline is intended to assist clinicians involved in the perioperative management of surgical patients requiring mechanical ventilation, including anesthesiologists, intensivists, and perioperative care teams. It covers intraoperative and immediate postoperative periods and includes general and specific recommendations for high-risk populations (e.g., obese, pediatric, thoracic or cardiac surgery patients). The working group was organized into thematic subgroups, each assigned to specific perioperative topics related to mechanical ventilation. Each subgroup consisted of two members with recognized expertise in the assigned topic. All members were selected based on their clinical, academic, or research experience in perioperative care.

#### Development process

Seventeen structured clinical questions were formulated using the PICO format (Population, Intervention, Comparison, Outcome), addressing key challenges in perioperative ventilatory care. Each question guided the systematic search, analysis, and synthesis of available evidence, as well as the development of the corresponding recommendations.

### Literature review and data sources

A comprehensive literature search was conducted using MEDLINE (via PubMed), Embase, the Cochrane Library, and Scopus. Searches focused on identifying randomized controlled trials, meta-analyses, observational studies, and high-quality reviews relevant to each PICO question. Manual reference tracking was also performed. Search strategies and date ranges were tailored for each topic, covering studies from database inception up to July 2025.

Detailed search strategies, inclusion and exclusion criteria, study selection processes, and risk of bias assessment methods are provided in the Supplementary Material.

### Assessment of evidence and GRADE classification

The quality of evidence and strength of recommendations were assessed using the GRADE (Grading of Recommendations, Assessment, Development and Evaluation) approach. The GRADE methodology considers the quality of evidence (high, moderate, low, or very low) and balances the benefits and harms of interventions. Each recommendation was explicitly labeled with its strength (strong or weak/conditional) and the corresponding level of evidence. Interpretation of conditional recommendations: in this guideline, weak/conditional recommendations with low or very low certainty of evidence should be interpreted as potentially useful strategies in selected patients or specific contexts, particularly in centers with expertise and available resources.^16^^,^^17^ The panel considered these interventions relevant to maintain as conditional options despite the limitations of the evidence. Detailed voting results and agreement rates for all recommendations are presented in Supplementary Table S1.

It should also be emphasized that, according to the GRADE framework, the strength of a recommendation does not always reflect the quality of evidence. In some circumstances, a strong recommendation may be warranted even with low or very low certainty (e.g., when the intervention has clear benefit, minimal risk, low cost, or lack of safe alternatives). Conversely, weak recommendations may arise despite moderate or high-quality evidence when uncertainties persist regarding risks, variability in patient values, or feasibility of implementation.

Risk of bias was assessed according to study design using established tools, and these assessments informed the certainty of evidence within the GRADE framework, as detailed in the Supplementary Material.

### Development of recommendations and consensus process

Each subgroup drafted initial recommendations based on the available evidence. These were refined through iterative rounds of review within the panel. Agreement was assessed using a modified Delphi process.^18^ When the predefined threshold of at least 80% agreement was not achieved, recommendations were discussed until consensus was reached. The final recommendations and supporting rationale were standardized and approved for inclusion in the guideline.

### Review and editorial standardization

A central editorial committee oversaw the final review, formatting, and standardization of the document. All chapters were structured uniformly, including background, clinical question (PICO), methodology, explicit recommendations with GRADE classification, rationale grounded in the literature, and a Summary Table.

### Conflict of interest and funding

All contributors disclosed potential conflicts of interest, which were managed in accordance with SBA policy. No commercial entities were involved at any stage of the guideline development or had any influence over its content.

## Results – Recommendations overview

### Definition of postoperative pulmonary complications in surgical patients

Postoperative Pulmonary Complications (PPCs) are major contributors to morbidity, mortality, prolonged hospitalization, and increased costs after surgery.^2^ A key limitation in care and research is the heterogeneity of definitions. Standardized criteria, applied within a prespecified time window, are essential for reproducible identification, inter-study comparability, and guiding preventive strategies. In this guideline, PPCs are assessed during the index admission or up to Day 7; major events (pneumonia, ARDS, unplanned reintubation, death) are also captured through Day 30. PPCs should be defined using standardized clinical, radiological, and laboratory criteria, aligned with EPCO (European Perioperative Clinical Outcome)^19^ and StEP (Standardised Endpoints in Perioperative Medicine).^20^^,^^21^ Pneumonia should follow CDC/NHSN definitions,^22^ and ARDS should be defined by the Berlin definition, acknowledging the New Global Definition, which extends Berlin (e.g., High-Flow Nasal Cannula and SpO_2_/FiO_2_) and may be applied when arterial blood gases are unavailable.^23^

#### PICO clinical question

In adult surgical patients (P), does using standardized definitions for postoperative pulmonary complications (I), compared with heterogeneous or non-standardized criteria (C), improve reproducibility and prognostic validity, enhance inter-study comparability, and facilitate implementation of preventive strategies (O)?

**Table:** Evidence-based recommendations.

Recommendation	GRADE Recommendation	Evidence Quality
PPCs should be defined using standardized clinical, radiological, and laboratory criteria.	Strong	High.^19^^,^^24^
Definitions should rely on objective parameters to allow reproducibility and comparability across studies.	Strong	Moderate.^25^^,^^26^
A core set of complications, such as respiratory failure, pleural effusion, pneumothorax, atelectasis, bronchospasm, aspiration pneumonitis, pneumonia, and ARDS should be routinely assessed.	Strong	High.^27^^,^^28^
Using harmonized definitions improves benchmarking, outcome tracking, and implementation of protective strategies.	Strong	Moderate.^2^^,^^29^

PPCs, Postoperative Pulmonary Complications; ARDS, Acute Respiratory Distress Syndrome; GRADE, Grading of Recommendations, Assessment, Development and Evaluation.

This core set should be applied consistently in clinical practice and research, while allowing for updates as new diagnostic tools become available.

#### Rationale and literature-based discussion

PPCs are prevalent and clinically relevant across surgical populations. Studies report an incidence of 2%–10% in general surgical patients and up to 40% in high-risk groups.^2^ Despite their significance, the absence of standardized definitions across studies impairs comparison and meta-analysis of results.

Several prospective studies and clinical guidelines have proposed diagnostic frameworks incorporating objective criteria such as PaO_2_/FiO_2_ ratios, SpO_2_ thresholds, radiographic findings, and treatment requirements (e.g., oxygen therapy, bronchodilators, or antibiotics).^12^^,^^19^, ^20^, ^21^ The use of unified criteria has enabled robust comparisons in large multicenter studies and trials, LAS VEGAS^30^ and iPROVE.^31^

Conditions such as pneumonia, atelectasis, and aspiration pneumonitis require specific diagnostic markers due to their pathophysiological and therapeutic implications. A structured diagnostic approach supports clinical decisions, guides ventilatory strategies, and enables integration into quality metrics in perioperative care. Although CDC National Healthcare Safety Network (NHSN) surveillance criteria are recommended for the definition of postoperative pneumonia, their application in the immediate postoperative period requires caution. Early radiographic abnormalities are common after surgery and often reflect atelectasis, residual anesthetic effects, or inflammatory changes rather than true infection. Therefore, in surgical patients, CDC/NHSN-based pneumonia definitions should be interpreted in conjunction with clinical evolution, systemic signs of infection, and microbiological data when available, to avoid misclassification of early postoperative pulmonary changes such as pneumonia (Summary Table 1)

**Summary Table 1:** Operational definitions of common postoperative pulmonary complications.

Complication	Operational definition	Key clinical note
Respiratory failure.^1^^,^^9^^,^^19^^,^^27^^,^^32^	Any one of: PaO_2_/FiO_2_ < 300; SpO_2_ ≤ 90% on room air with need for supplemental O_2_; unplanned NIV/HFNC or invasive ventilation; unplanned reintubation for a respiratory cause; hypercapnic failure with pH < 7.30 attributable to ventilatory pump failure.	Core outcome; associated with longer length of stay, ICU admission, and higher mortality.
Pleural effusion.^19^^,^^27^^,^^32^, ^33^, ^34^	Lung ultrasound showing a mobile anechoic collection or Chest X-Ray (CXR) with meniscus/costophrenic blunting in a compatible context.	Common after thoracic/cardiac surgery; may worsen hypoxemia and delay recovery.
Pneumothorax.^1^^,^^3^^,^^9^^,^^19^^,^^27^^,^^32^, ^33^, ^34^.	CXR with a visceral pleural line and absent distal lung markings, or lung ultrasound with absent lung sliding and barcode/stratosphere sign on M-mode (lung point ‒ when present ‒ is highly specific).	Prompt recognition prevents deterioration; may follow positive-pressure barotrauma or central line insertion.
Atelectasis.^8^^,^^9^^,^^19^^,^^27^^,^^32^, ^33^, ^34^	Opacity with volume loss (fissure/mediastinal shift or hemidiaphragm elevation), segmental or lobar.	Most frequent PPC; anesthesia-induced collapse; contributes to hypoxemia.
Bronchospasm.^8^^,^^9^^,^^19^^,^^27^^,^^32^, ^33^, ^34^	New expiratory wheeze requiring bronchodilator therapy with clinical/ventilatory improvement.	Frequent in asthma/COPD; may complicate extubation.
Aspiration pneumonitis.^1^^,^^9^^,^^19^^,^^27^^,^^32^^,^^33^	Acute lung injury after witnessed/suspected gastric aspiration with a new infiltrate within 2–24 hours and hypoxemia.	Preventable with airway protection; can progress to ARDS.
Pneumonia.^3^^,^^9^^,^^10^^,^^19^^,^^32^, ^33^, ^34^	Preferred: CDC/NHSN surveillance criteria (NV-HAP/VAP).^22^ Alternative (where surveillance is not feasible): CXR new/progressive infiltrate plus ≥ 2 of fever, leukocytosis/leukopenia, purulent secretions, or worsening oxygenation. Specify which criterion was used.	Increases morbidity and mortality; enables benchmarking when CDC/NHSN is used.
Acute respiratory distress syndrome (ARDS).^23^	**Global Definition of ARDS (2023):** bilateral opacities on CXR/CT or lung ultrasound by a trained professional; onset ≤ 1 week after a known risk; not explained by cardiogenic edema/overload. **Hypoxemia ‒ non‑intubated:** HFNC ≥ 30 L.min^-1^ or CPAP/NIV with PEEP ≥ 5 cm H_2_O, and S/F ≤ 315 measured with SpO_2_ ≤ 97%. **Severity**: mild, 200 < P/F ≤ 300 or 235 < S/F ≤ 315; moderate, 100 < P/F ≤ 200 or 148 < S/F ≤ 235; severe, P/F ≤ 100 or S/F ≤ 148 .	Severe but less common; requires ICU-level management.

PaO_2_, Arterial Oxygen tension; FiO_2_, Fraction of inspired oxygen; SpO_2_, Peripheral Oxygen saturation; O_2_, Oxygen; NIV, Non-Invasive Ventilation; HFNC, High-Flow Nasal Cannula; ICU, Intensive Care Unit; CXR, Chest X-Ray; CT, Computed Tomography; COPD, Chronic Obstructive Pulmonary Disease; CDC, Centers for Disease Control and Prevention; NHSN, National Healthcare Safety Network; NV-HAP, Non-Ventilator Hospital-Acquired Pneumonia; VAP, Ventilator-Associated Pneumonia; ARDS, Acute Respiratory Distress Syndrome; CPAP, Continuous Positive Airway Pressure; PEEP, Positive End-Expiratory Pressure; S/F, SpO_2_/FiO_2_ ratio; P/F, PaO_2_/FiO_2_ ratio.

### Postoperative pulmonary complications: risk factors and preventive strategies

Postoperative Pulmonary Complications (PPCs) are a significant cause of increased morbidity, mortality, and healthcare costs in surgical patients.^29^ Early identification of risk factors, especially modifiable ones, is essential to guide preventive strategies and improve perioperative respiratory care, ultimately reducing the incidence of PPCs across surgical settings.

#### PICO clinical question

In adult surgical patients (P), does identifying and addressing perioperative risk factors (I), compared to not managing such risk factors (C), reduce the incidence of postoperative pulmonary complications (O)?

**Table:** Evidence-based recommendations.

Recommendation	**GRADE Recommendation**	**Evidence Quality**
Preoperative risk stratification using validated tools (e.g., ARISCAT score) is recommended to identify patients at high risk for PPCs.	Strong	High.^30^^,^^35^
Optimization of comorbidities (e.g., COPD, heart failure, malnutrition, anemia) should be undertaken before surgery whenever possible.	Strong	Moderate.^36^^,^^37^
Smoking cessation should be recommended at least 4 weeks before surgery, ideally 4–8 weeks, to reduce the risk of PPCs.	Strong	Moderate.^38^^,^^39^
Supervised respiratory physiotherapy and early mobilization, including prehabilitation for thoracic/cardiac surgery and structured postoperative therapy in high-risk patients, may be considered.	Weak	Moderate.^40^^,^^41^
Minimally invasive approaches (e.g., laparoscopy) are associated with reduced PPCs when feasible; however, surgical positioning and pneumoperitoneum-related effects should be considered in high-risk patients.	Strong	Moderate.^42^^,^^43^
Bundle-based preventive strategies (multimodal perioperative protocols) are encouraged to maximize impact on reducing PPCs.	Strong	Moderate.^28^^,^^44^

PPCs, Postoperative Pulmonary Complications; COPD, Chronic Obstructive Pulmonary Disease; GRADE, Grading of Recommendations, Assessment, Development and Evaluation.

#### Rationale and literature-based discussion

Identification and mitigation of risk factors are crucial in reducing PPCs. Several patient-related factors, such as advanced age, obesity, pre-existing lung disease, smoking, and poor functional status are strongly associated with increased risk. Surgical factors including prolonged operative time, upper abdominal or thoracic incisions, and emergency procedures further increase susceptibility.^32^

The ARISCAT score^35^ has been widely validated as a practical tool for predicting PPCs, incorporating variables such as age, preoperative SpO_2_, type of surgery, and duration. Although not perfect, its use provides a structured framework for preoperative risk stratification.

COPD optimization with bronchodilators, correction of anemia, and nutritional support are associated with improved outcomes. Smoking cessation has consistently been linked to a reduction in PPCs, with greater benefit observed when cessation occurs at least four weeks before surgery.

Non-pharmacologic strategies, including incentive spirometry, chest physiotherapy, and early mobilization, are recommended in high-risk populations, although evidence is heterogeneous. Surgical approaches also play a major role; laparoscopic and minimally invasive techniques are associated with lower PPC incidence compared with open procedures.

Finally, multimodal bundles that integrate risk stratification, optimization, and intra- and postoperative strategies have shown the greatest impact in reducing PPCs, underscoring the need for integrated perioperative care.

**Table:** Summary Table Risk Factors and Prevention of PPCs.

Domain	Key Strategies	Clinical Considerations
Risk Stratification	Use ARISCAT or other validated tools	Identify patients requiring tailored perioperative strategies
Comorbidities	Optimize COPD, CHF, malnutrition, anemia	Improve perioperative resilience and recovery
Smoking	Cessation ≥ 4–8 weeks before surgery	Reduce PPC risk; counseling should be offered
Non-pharmacologic interventions	Supervised respiratory physiotherapy and early mobilization; include prehabilitation when feasible	Beneficial in high-risk patients; evidence is heterogeneous
Surgical approach	Favor minimally invasive surgery when feasible	Associated with lower incidence of PPCs
Bundles	Apply multimodal protocols	Greater effectiveness than isolated interventions

ARISCAT, Assess Respiratory Risk in Surgical Patients in Catalonia; COPD, Chronic Obstructive Pulmonary Disease; CHF, Congestive Heart Failure; PPCs, Postoperative Pulmonary Complications.

### Modes and modalities of invasive mechanical ventilation during the intraoperative period

The choice of ventilation mode can influence intraoperative gas exchange, pulmonary mechanics, hemodynamics, and the risk of postoperative pulmonary complications.^42^ Understanding the application and physiological implications of different ventilation modes in the operating room is key to implementing lung-protective ventilation strategies tailored to surgical patients.

#### PICO clinical question

In adult surgical patients under general anesthesia (P), do different ventilatory modes (I), compared to other available modes (C), reduce intraoperative complications and improve gas exchange or respiratory mechanics (O)?

**Table:** Evidence-based recommendations.

**Recommendation**	**GRADE Recommendation**	**Evidence Quality**
Volume-Controlled Ventilation (VCV) and Pressure-Controlled Ventilation (PCV) provide comparable outcomes when protective settings are applied.	Strong	Moderate.^45^, ^46^, ^47^
Dual-control modes (e.g., PCV-VG) may be considered to optimize tidal volume delivery in patients with variable compliance, particularly in obesity and laparoscopy.	Weak	Low.^48^^,^^49^
Use pressure-controlled ventilation when peak airway pressures are a concern, recognizing that this strategy may limit peak pressure but at the expense of tidal volume delivery.	Weak	Low.^45^^,^^50^
Avoid high-frequency oscillatory ventilation or unconventional modes intraoperatively outside research settings.	Strong	Moderate.^51^^,^^52^
In patients with compromised respiratory mechanics, individualized mode selection is encouraged.	Strong	Low.^53^^,^^54^

VCV, Volume-Controlled Ventilation; PCV, Pressure-Controlled Ventilation; PCV-VG, Pressure-Controlled Ventilation with Volume Guarantee; GRADE, Grading of Recommendations, Assessment, Development and Evaluation.

#### Rationale and literature-based discussion

The two most used ventilation modes during surgery are Volume-Controlled Ventilation (VCV) and Pressure-Controlled Ventilation (PCV). Both allow for the application of protective strategies with low tidal volumes, moderate PEEP, and monitoring of driving pressure.

Studies comparing VCV and PCV show similar clinical outcomes when protective parameters are maintained. However, PCV may reduce peak inspiratory pressures due to its decelerating flow, which is beneficial in patients with reduced pulmonary compliance (e.g., obesity, laparoscopic procedures).

PCV with Volume Guarantee (PCV-VG) combines the benefits of both VCV and PCV, ensuring target tidal volumes while adapting pressure delivery. This mode is increasingly used in intraoperative settings with variable compliance.

Despite theoretical benefits, there is no strong evidence that one ventilation mode is superior to another in reducing postoperative pulmonary complications if protective settings are properly applied.

**Table:** **Summary Table** Ventilation modes and intraoperative considerations.

**Mode**	**Characteristics**	**Considerations**
Volume-Controlled (VCV)	Constant flow, fixed tidal volume	Reliable delivery of volume; may increase peak pressure in low compliance lungs
Pressure-Controlled (PCV)	Decelerating flow, pressure-targeted	Reduces peak pressure; tidal volume varies with compliance/resistance
PCV-VG (dual control)	Pressure-targeted, volume guaranteed	Combines benefits of VCV and PCV; useful in obesity/laparoscopy
Advanced/Unconventional	Includes HFOV, PAV, NAVA	Limited role intraoperatively; not routinely recommended

VCV, Volume-Controlled Ventilation; PCV, Pressure-Controlled Ventilation; PCV-VG, Pressure-Controlled Ventilation with Volume Guarantee; HFOV, High-Frequency Oscillatory Ventilation; PAV, Proportional Assist Ventilation; NAVA, Neurally Adjusted Ventilatory Assist.

### Basic monitoring of intraoperative mechanical ventilation

Intraoperative monitoring of mechanical ventilation is a fundamental aspect of implementing protective ventilation strategies and minimizing PPCs. Basic respiratory mechanics monitoring techniques such as pressure and flow curves, loops, and specific pressure measurements (plateau, peak, driving, and transpulmonary pressure) provide critical real-time feedback to individualize mechanical ventilation and reduce VILI.

#### PICO clinical question

In adult surgical patients under general anesthesia (P), does intraoperative monitoring of basic ventilatory parameters such as pressure-time and flow-time curves and loops (I), compared to no structured monitoring (C), reduce postoperative pulmonary complications (O)?

**Table:** Recommendations and Quality of Evidence (GRADE).

**Recommendation**	**GRADE Recommendation**	**Evidence Quality**
Suggest monitoring the pressure-time curve to detect overdistension or alveolar recruitment using the stress index (SI ≈ 1).	Weak	Low.^55^^,^^56^
Recommend using the flow-time curve to detect auto-PEEP, especially in high respiratory rate situations.	Strong	Very Low.^57^^,^^58^
Pressure-volume and flow-volume loops may aid in assessing compliance and resistance.	Weak	Very Low.^59^^,^^60^
Recommend monitoring plateau pressure and maintaining it below 25–30 cm H_2_O.	Strong	Very Low.^61^^,^^62^
Suggest interpreting plateau pressure with caution in cases of altered chest wall mechanics.	Weak	Very Low.^63^
Transpulmonary pressure monitoring (via esophageal balloon) may be useful in individualizing PEEP in complex cases, provided expertise and equipment are available.	Weak	Very Low.^63^
Recommend monitoring driving pressure (ΔP) aiming for values ≤ 15 cm H_2_O.	Strong	Moderate.^64^, ^65^, ^66^
Mechanical power monitoring may help adjust ventilatory parameters to reduce PPCs.	Weak	Low.^67^^,^^68^

SI, Stress Index; auto-PEEP, Intrinsic Positive End-Expiratory Pressure; PEEP, Positive End-Expiratory Pressure; ΔP, Driving Pressure; PPCs, Postoperative Pulmonary Complications; GRADE, Grading of Recommendations, Assessment, Development and Evaluation.

#### Rationale and literature-based discussion

##### Pressure-time curve

**Figure 2:**
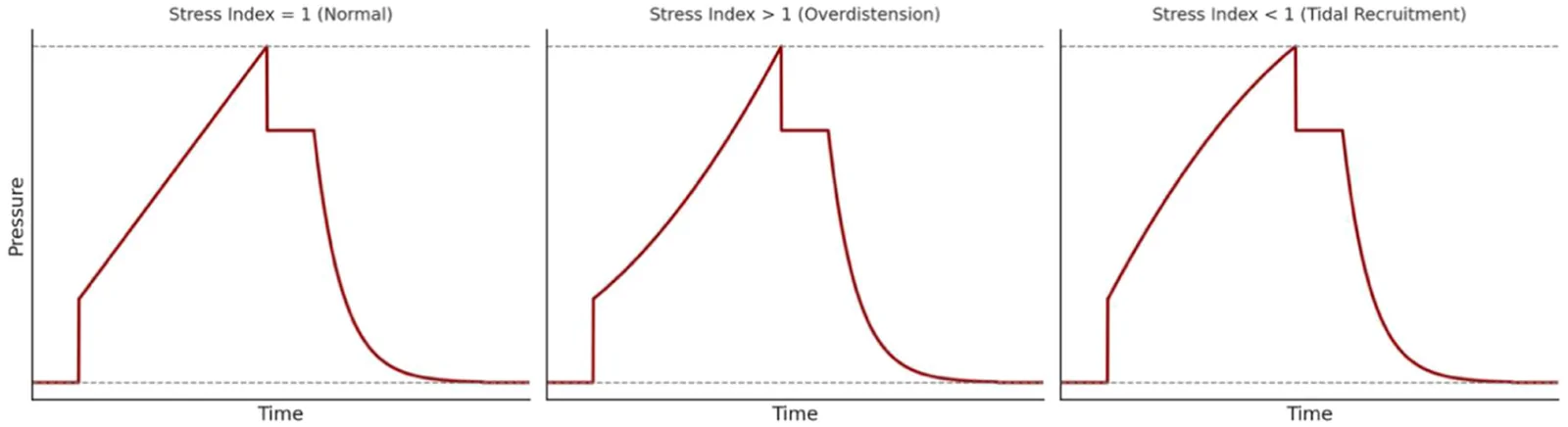
Pressure-time curve during volume-controlled mechanical ventilation.

The pressure-time curve helps evaluate dynamic compliance and alveolar behavior during the inspiratory phase. A linear slope (SI = 1) indicates stable compliance, while SI > 1 suggests overdistension and SI < 1 indicates recruitment. Adjusting tidal volume or PEEP based on the stress index may reduce VILI (Fig. 2).

##### Flow-time curve

Failure of expiratory flow to return to zero before the next breath suggests the presence of auto-PEEP, often caused by insufficient exhalation time or high respiratory rate. Auto-PEEP increases the risk of dynamic hyperinflation and hemodynamic instability.

##### Pressure-volume and flow-volume loops

Pressure-volume loops provide a visual understanding of compliance changes and inflection points for alveolar opening and overdistension. Flow-volume loops help detect airway obstruction or leaks, especially in the presence of bronchospasm, secretion accumulation, or ventilator circuit issues.

##### Peak pressure

Although nonspecific, an increase in peak pressure may reflect increased airway resistance. It should be interpreted alongside plateau pressure to distinguish elastic versus resistive contributions.

##### Plateau pressure

Obtained via an inspiratory pause, plateau pressure reflects alveolar pressure and should be kept ≤ 30 cm H_2_O to minimize overdistension. Its interpretation must consider conditions like obesity or pneumoperitoneum that increase pleural pressures.

##### Transpulmonary pressure (P_L)

Estimated using esophageal pressure, it reflects the distending force on the lung. While promising for tailoring PEEP, its role remains limited in routine intraoperative care due to technical complexity and lack of strong evidence in non-injured lungs.

##### Driving Pressure (ΔP)

Defined as plateau pressure minus PEEP or as VT/compliance, it is a key predictor of PPCs. Lower ΔP is associated with improved outcomes, making it a valuable target in intraoperative ventilatory strategy.

##### Mechanical power

Mechanical Power (MP): quantifies the energy delivered to the lungs per minute, integrating Vt, RR, PEEP, and ΔP. Higher MP is associated with increased VILI/PPC risk; however, clinical uptake is limited not only by complexity but primarily by the absence of RCTs and validated targets to guide adjustments.

**Table:** **Summary Table** Monitoring parameters and clinical use.

**Parameter**	**Clinical Application**	**Suggested Target**
Pressure-Time Curve	Identifies overdistension or recruitment (via Stress Index)	SI ≈ 1
Flow-Time Curve	Detects auto-PEEP and dynamic hyperinflation	Flow returns to zero before next breath
Pressure-Volume Loop	Assesses compliance and alveolar opening/overdistension	Visual inspection
Flow-Volume Loop	Identifies airway obstruction or leakage	Visual inspection
Peak Pressure	Indicates resistance (e.g., tube kinking, bronchospasm)	< 35–40 cm H_2_O
Plateau Pressure	Reflects alveolar pressure, risk of overdistension	≤ 25–30 cm H_2_O
Transpulmonary Pressure	Guides individualized PEEP (if available)	Based on esophageal pressure (Pes)
Driving Pressure (ΔP)	Strong predictor of PPCs, correlates with lung stress	≤ 15 cm H_2_O
Mechanical Power	Represents total ventilator-induced lung energy	Keep as low as possible, no universal threshold. Use trends to trigger reassessment of Vt (PBW), RR, PEEP, and flow/Ti.

SI, Stress Index; auto-PEEP, Intrinsic Positive End-Expiratory Pressure; PEEP, Positive End-Expiratory Pressure; Pes, Esophageal Pressure; ΔP, Driving Pressure; PPCs, Postoperative Pulmonary Complications; Vt, Tidal Volume; PBW, Predicted Body Weight. For practical bedside application, the predicted body weight calculation formula and a simplified height-based reference table are provided in the Supplementary Material (Supplementary Appendix S1); RR, Respiratory Rate; Ti, Inspiratory Time.

### Advanced monitoring of intraoperative mechanical ventilation

Technological advances in perioperative care have expanded the tools available for individualized monitoring of mechanical ventilation. Novel imaging and electrical techniques, such as Lung Ultrasound (LUS) and Electrical Impedance Tomography (EIT) allow real-time evaluation of alveolar recruitment, ventilation distribution, and the impact of ventilatory settings. These tools aim to enhance ventilatory support, optimize gas exchange, and minimize PPCs, particularly in high-risk populations undergoing laparoscopic or thoracic procedures.^69^^,^^70^

#### PICO clinical question

In adult surgical patients under general anesthesia and mechanical ventilation (P), do advanced monitoring modalities such as lung ultrasound or electrical impedance tomography (I), compared to standard monitoring (C), improve ventilatory accuracy and reduce postoperative pulmonary complications (O)?

**Table:** Evidence-Based Recommendations.

**Recommendation**	**GRADE Recommendation**	**Evidence Quality**
Use of lung ultrasound (LUS) to guide alveolar recruitment is suggested in laparoscopic or thoracoscopic surgery.	Strong	Moderate.^70^, ^71^, ^72^
EIT may be used intraoperatively to titrate PEEP and improve oxygenation and compliance, where available.	Weak	Moderate.^69^^,^^73^^,^^73^

LUS, Lung Ultrasound; EIT, Electrical Impedance Tomography; PEEP, Positive End-Expiratory Pressure; GRADE, Grading of Recommendations, Assessment, Development and Evaluation.

#### Rationale and literature-based discussion

##### Lung ultrasound (LUS)

Atelectasis is common during general anesthesia, especially in patients undergoing laparoscopic or Trendelenburg procedures. Several RCTs demonstrate that LUS-guided recruitment improves lung aeration, reduces driving pressure, and decreases PPCs when compared to conventional maneuvers:

• Lung ultrasound-guided recruitment consistently improved oxygenation, aeration, and reduced atelectasis compared with standard strategies across randomized trials in laparoscopic and thoracoscopic surgery.^70^, ^71^, ^72^

##### Electrical Impedance Tomography (EIT)

EIT is a non-invasive, bedside, radiation-free imaging modality that monitors ventilation in real time. Its intraoperative use allows for individualized PEEP titration:

• Meta-analyses consistently demonstrated that EIT-guided PEEP optimization improves oxygenation, enhances compliance, and reduces driving pressure in surgical patients.^74^^,^^69^^,^^73^

These modalities provide dynamic functional insights and can be integrated into intraoperative decision-making to optimize mechanical ventilation in real time.

**Table:** **Summary Table** Applications and benefits of advanced monitoring techniques.

**Modality**	**Indication**	**Main Benefits**
Lung Ultrasound (LUS)	Laparoscopic/Thoracoscopic surgeries	Improves aeration, reduces driving pressure, and PPCs reduction reported in selected subgroups only.
Electrical Impedance Tomography (EIT)	General surgeries requiring PEEP titration	Enhances oxygenation, improves compliance, lowers ΔP

LUS, Lung Ultrasound; EIT, Electrical Impedance Tomography; PEEP, Positive End-Expiratory Pressure; ΔP, Driving Pressure; PPCs, Postoperative Pulmonary Complications.

### Noninvasive mechanical ventilation and high-flow nasal cannula in the perioperative period

Non-invasive respiratory support has become an important adjunct in perioperative care, particularly in patients at risk of hypoxemia or postoperative pulmonary complications.^75^ Non-Invasive Ventilation (NIV) improves gas exchange and reduces respiratory effort through positive airway pressure, while High-Flow Nasal Cannula (HFNC) delivers heated, humidified oxygen at high flows, providing low levels of positive pressure and enhanced comfort. Evidence shows that both modalities can be beneficial as preoxygenation strategies in high-risk patients, as selective intraoperative tools, and especially in the postoperative period, where NIV reduces reintubation and pneumonia and HFNC improves oxygenation and tolerance.^76^

#### PICO clinical question

In adult surgical patients (P), does the use of Non-Invasive Ventilation (NIV) or high-flow nasal cannula (HFNC) in the perioperative period (I), compared with conventional oxygen therapy (C), reduce hypoxemia, reintubation, and postoperative pulmonary complications (O)?

**Table:** Evidence-Based Recommendations.

**Recommendation**	**GRADE Recommendation**	**Evidence Quality**
NIV is recommended for the treatment of acute postoperative hypoxemic respiratory failure in cooperative, hemodynamically stable patients.	Strong	High.^77^^,^^78^
HFNC is recommended as an alternative to conventional oxygen therapy after extubation in high-risk surgical patients to improve oxygenation and comfort.	Strong	Moderate.^79^^,^^80^
NIV or HFNC may be considered for preoxygenation before intubation in obese or hypoxemic patients to prolong safe apnea time and reduce desaturation.	Weak	Moderate.^76^^,^^81^
NIV is not recommended in patients with contraindications such as airway obstruction, hemodynamic instability, impaired consciousness, or copious secretions.	Strong	Moderate.^82^^,^^83^
Routine prophylactic use of NIV in unselected postoperative patients is not recommended, especially after abdominal or thoracic surgery.	Strong	High.^84^^,^^85^
HFNC may be considered in patients intolerant to NIV interfaces or when NIV is contraindicated.	Weak	Moderate.^86^^,^^87^

NIV, Non-Invasive Ventilation; HFNC, High-Flow Nasal Cannula; GRADE, Grading of Recommendations, Assessment, Development and Evaluation.

#### Rationale and literature-based discussion

Evidence synthesized up to 2025 confirms the important role of NIV and HFNC in perioperative care.^75^ Preoperatively, both modalities can improve preoxygenation and extend safe apnea time, particularly in obese or hypoxemic patients, with HFNC often better tolerated. Intraoperatively, their role is limited to specific scenarios under sedation or regional anesthesia where intubation is undesirable. Postoperatively, NIV has the strongest evidence, significantly reducing reintubation, pneumonia, and ICU stay in acute hypoxemic respiratory failure, while HFNC improves oxygenation and comfort in high-risk patients, although with less consistent outcome data. Routine prophylactic use in unselected patients is not recommended, and both modalities should never delay timely intubation. Preoxygenation with NIV or HFNC enhances oxygen reserve and prolongs safe apnea time during induction, especially in obese patients, those with reduced FRC, or severe hypoxemia.^81^

**Table:** **Summary Table** Perioperative use of NIV and HFNC.

**Perioperative Phase**	**Evidence-Based Application**	**Clinical Considerations**
Preoperative	Effective for preoxygenation in obese/hypoxemic patients; prolongs safe apnea.	HFNC may be better tolerated; not required for all patients.
Intraoperative	Option in selected non-intubated patients under regional anesthesia or sedation.	Limited evidence requires close monitoring.
Postoperative	NIV reduces reintubation and pneumonia in acute hypoxemia; HFNC effective after extubation in high-risk patients.	Avoid routine prophylaxis; do not delay intubation in case of failure.

NIV, Non-Invasive Ventilation; HFNC, High-Flow Nasal Cannula.

### Intraoperative mechanical ventilation in patients with or without acute lung injury

Intraoperative mechanical ventilation has evolved from a secondary anesthetic concern to a cornerstone of perioperative care. While previously based on high tidal volumes and minimal PEEP, it is now well established that non-protective ventilation strategies, even during short-term surgery can cause VILI and increase the risk of PPCs.^88^^,^^89^ Both patients with healthy lungs and those with Acute Lung Injury (ALI) benefit from ventilation strategies tailored to minimize pulmonary stress and improve clinical outcomes.^90^

#### PICO clinical question

In adult surgical patients with or without acute lung injury (P), do lung-protective ventilatory strategies (I), compared to conventional ventilation (C), reduce the risk of postoperative pulmonary complications and ventilator-induced lung injury (O)?

**Table:** Evidence-Based Recommendations.

**Recommendation**	**GRADE Recommendation**	**Evidence Quality**
Use tidal volumes of 6–8 mL.kg-1 PBW in surgical patients without ARDS	Strong	High.^88^^,^^91^
Avoid tidal volumes > 10 gt; 10 mL.kg^-1^ PBW.	Strong	High.^88^^,^^92^
Maintain plateau pressure ≤ 30 lt; 30 cm H_2_O.	Strong	High.^88^^,^^90^
Target driving pressure ≤ 15 lt; 15 cm H_2_O, ideally ≤ 12 cm H_2_O.	Strong	Moderate.^91^^,^^93^
Use individualized PEEP titrated to oxygenation and respiratory mechanics (ΔP/compliance); avoid routine ZEEP* in higher-risk contexts (e.g., obesity, pneumoperitoneum/Trendelenburg).	Strong	Moderate.^90^^,^^94^
Favor early extubation in stable patients.	Strong	High.^55^^,^^93^
Consider individualized PEEP guided by physiological signals (e.g., esophageal pressure or EIT) in higher-risk settings (obesity, pneumoperitoneum/Trendelenburg) to optimize oxygenation and mechanics; evidence for reducing PPCs remains.	Weak	Moderate.^90^^,^^95^

PBW, Predicted Body Weight. For practical bedside application, the predicted body weight calculation formula and a simplified height-based reference table are provided in the Supplementary Material; PEEP, Positive End-Expiratory Pressure; ΔP, Driving Pressure; ZEEP, Zero End-Expiratory Pressure; EIT, Electrical Impedance Tomography; PPCs, Postoperative Pulmonary Complications; GRADE, Grading of Recommendations, Assessment, Development and Evaluation.

#### Rationale and literature-based discussion

Protective ventilation strategies minimize alveolar overdistension and atelectasis. In patients with healthy lungs, high tidal volumes increase transpulmonary pressures and can precipitate VILI. Trials such as PROVHILO and iPROVE^88^, ^96^ have shown that low tidal volumes (6–8 mL.kg^-1^ PBW) reduce PPCs and improve recovery.

For patients with acute lung injury, intraoperative ventilatory care should mimic ARDS management in the ICU, focusing on low plateau pressures (≤ 30 cm H_2_O) and driving pressure reduction, with data from Amato et al.^97^ highlighting the strong predictive value of driving pressure for mortality.

PEEP remains essential to counteract intraoperative atelectasis, especially in at-risk populations such as the obese and those undergoing laparoscopy. While PEEP < 5 cm H_2_O is inadequate, individualized titration based on compliance or driving pressure may enhance safety and efficacy. Esophageal pressure monitoring and EIT are useful tools where available.

Respiratory rate should be titrated to avoid hypercapnia and maintain PaCO_2_ between 38–43 mmHg.^55^^,^^94^ In all cases, early extubation when feasible, and avoiding hyperoxia, are important for enhancing outcomes.^55^^,^^96^ PEEP: Use individualized PEEP titrated to oxygenation and respiratory mechanics (ΔP/compliance); avoid routine ZEEP in higher-risk contexts (e.g., obesity, pneumoperitoneum/Trendelenburg).

**Table:** Summary Table Recommended intraoperative ventilation strategies.

**Parameter**	**Recommended Strategy**	**Clinical Considerations**
Tidal Volume	6–8 mL.kg^-1^ PBW; avoid tidal volumes > 10 mL.kg^-1^ PBW	Low tidal volumes reduce the risk of VILI and PPCs. Use 6–8 mL·kg⁻¹ PBW in patients without ARDS. In patients with ARDS, use 4–8 mL·kg⁻¹ PBW, adjusted to maintain lung protective pressure targets.
Plateau Pressure (Pplat)	Maintain ≤ 30 cm H_2_O	Prevents alveolar overdistension; critical in patients with ALI/ARDS.
Driving Pressure (ΔP)	Keep ≤ 15 cm H_2_O	Strong predictor of mortality; minimizing ΔP is more protective than VT alone.
PEEP	≥ 5 cmH₂O in all patients; individualized in ALI/obese	Counters atelectasis; titration based on compliance or ΔP is preferable.
Respiratory Rate	Adjust to maintain PaCO_2_ 38–43 mmHg	Prevents hypercapnia and ensures adequate minute ventilation.
FiO₂	Use lowest FiO_2_ to keep SpO_2_ 92%–97%	Avoids both hypoxemia and oxygen toxicity.
Extubation	Favor early extubation in stable patients	Reduces PPCs and facilitates recovery.
Advanced Monitoring	Consider esophageal-pressure monitoring or EIT in obesity / ALI/ Pneumoperitoneum / Trendelenburg to individualize PEEP; use in trained teams.	Useful for individualized PEEP and improved safety.

PBW, Predicted Body Weight. For practical bedside application, the predicted body weight calculation formula and a simplified height-based reference table are provided in the Supplementary Material; VILI, Ventilator-Induced Lung Injury; PPCs, Postoperative Pulmonary Complications; ARDS, Acute Respiratory Distress Syndrome; ALI, Acute Lung Injury; Pplat, Plateau Pressure; ΔP, Driving Pressure; PEEP, Positive End-Expiratory Pressure; PaCO_2_, Arterial Carbon Dioxide tension; FiO_2_, Fraction of inspired oxygen; SpO_2_, Peripheral Oxygen saturation; EIT, Electrical Impedance Tomography.

### Intraoperative pulmonary recruitment maneuvers

Intraoperative pulmonary Recruitment Maneuvers (RMs) are designed to reverse anesthesia-induced atelectasis and optimize gas exchange during mechanical ventilation.^89^^,^^90^ Despite their potential benefits, such as improved oxygenation and lung compliance, these techniques may carry risks, particularly hemodynamic compromise. Therefore, RMs should be applied selectively and based on individualized patient assessments and physiologic responses.

#### PICO clinical question

In adult surgical patients under general anesthesia (P), do intraoperative recruitment maneuvers (I), compared to no recruitment maneuvers (C), improve oxygenation, lung compliance, and reduce postoperative pulmonary complications (O)?

**Table:** Evidence-Based Recommendations (GRADE).

**Recommendation**	**GRADE Recommendation**	**Evidence Quality**
Use recruitment maneuvers in combination with low tidal volume ventilation and individualized PEEP in high-risk patients	Weak	Moderate.^88^^,^^94^
Apply recruitment maneuvers in cases of intraoperative hypoxemia or suspected reversible atelectasis	Weak	Moderate.^98^^,^^99^
Avoid routine high-pressure RMs (≥ 40 cm H_2_O for 30s) in all patients, especially without hemodynamic monitoring	Strong	Moderate.^55^^,^^100^

RM, Recruitment Maneuver; PEEP, Positive End-Expiratory Pressure; GRADE, Grading of Recommendations, Assessment, Development and Evaluation.

#### Rationale and literature summary

Intraoperative atelectasis is common during general anesthesia as functional residual capacity decreases, the diaphragm shifts cephalad, and dependent airways close. Recruitment Maneuvers (RMs) are intended to reverse atelectasis and improve gas exchange. Stepwise, physiology-guided approaches (e.g., graded PEEP increases followed by titration) are generally better tolerated hemodynamically than sustained inflations. A Bayesian individual-patient-data meta-analysis^98^ pooling PROVHILO,^101^ iPROVE,^100^ and PROBESE^102^ suggests a moderate reduction in PPCs when higher-PEEP strategies combined with RMs are embedded within lung-protective ventilation, with a clearer signal in laparoscopic surgery. By contrast, the parent trials (PROVHILO^101^; PROBESE^102^) reported more intraoperative hypotension and no consistent improvement in hard outcomes in unselected populations. Consistent with additional recent syntheses,^99^ we recommend that RMs be applied selectively, triggered by intraoperative hypoxemia or suspected reversible atelectasis and integrated with low tidal volumes and individualized PEEP, with appropriate hemodynamic monitoring.

**Table:** **Summary Table** Intraoperative recruitment maneuvers.

**Technique**	**Mechanism / Application**	**Benefits**	**Risks / Limitations**
Stepwise PEEP Recruitment	Incremental PEEP increase followed by titration	Better compliance, oxygenation	Requires hemodynamic monitoring
Sustained Inflation	Continuous pressure (30–40 cm H_2_O for 30–40s)	Atelectasis reversal	Hypotension, not well tolerated in all patients
Cyclic / Periodic RM	Applied every 30–60 min or after disconnections	Maintains aeration during long procedures	Limited data on long-term outcomes
RM + Decremental PEEP Titration	RM followed by PEEP titration to optimize oxygenation/compliance	May individualize PEEP	Requires specialized monitoring and experience

RM, Recruitment Maneuver; PEEP, Positive End-Expiratory Pressure.

### Hypoxemia and hyperoxia in surgical patients

Hypoxemia is a frequent postoperative complication and has been associated with increased morbidity and mortality.^103^ Conversely, excessive oxygen administration, especially in the absence of hypoxemia, may contribute to oxidative stress, atelectasis, and worsened clinical outcomes.^104^ Current evidence suggests that avoiding both extremes, hypoxemia and hyperoxia, is essential to optimizing patient safety. A balanced and individualized approach to oxygen therapy in surgical patients is strongly recommended.

To facilitate bedside decision making, a concise stepwise algorithm for the practical management of perioperative hypoxemia is provided in the Supplementary Material (Supplementary Appendix S2).

#### Clinical question (PICO)

In adult surgical patients (P), does targeting intraoperative and postoperative peripheral oxygen saturation (SpO_2_) between 92%–97% (I), compared to values below 92% or above 97% (C), reduce postoperative pulmonary complications and oxygen-related adverse outcomes (O)?

**Table:** Evidence-Based Recommendations.

**Recommendation**	**GRADE Recommendation**	**Evidence Quality**
Maintain SpO_2_ between 92% and 97% during the perioperative period.	Weak	Moderate.^105^^,^^106^
Avoid liberal oxygen therapy (SpO_2_ ≥ 98%) in the absence of hypoxemia.	Weak	Moderate.^107^^,^^108^
Avoid targeting SpO_2_ < 90% intra- and postoperatively due to the increased risk of mortality.	Strong	Moderate.^104^^,^^109^

SpO_2_, Peripheral Oxygen saturation; GRADE, Grading of Recommendations, Assessment, Development and Evaluation.

#### Rationale and literature-based discussion

Perioperative hypoxemia is often multifactorial and may result from atelectasis, ventilation perfusion mismatch, residual anesthetic effects, patient positioning, pneumoperitoneum, or hemodynamic alterations. A structured and systematic approach is therefore essential to rapidly identify reversible causes and guide timely corrective interventions. For the purpose of this guideline, perioperative hypoxemia is operationally defined as a peripheral oxygen saturation (SpO₂) below 90%, a threshold consistently associated with adverse clinical outcomes in observational and interventional studies.

Postoperative hypoxemia (SpO_2_ < 90%) is commonly observed in patients recovering from general anesthesia and is strongly associated with adverse outcomes, including increased mortality at 30 days and 1-year. Large cohort studies have reported that even transient episodes of severe desaturation are linked to ischemic events and increased healthcare utilization.^110^

On the other hand, liberal oxygen strategies using high inspired oxygen fractions (FiO_2_ ≥ 0.8) or targeting SpO_2_ ≥ 98% may increase the risk of oxygen toxicity, absorption atelectasis, and pulmonary inflammation. Trials such as the Oxygen-ICU and HYPERS2S demonstrated worse clinical outcomes in patients exposed to hyperoxia.^104^^,^^111^ Conservative oxygen strategies, as explored in the ICU-ROX and HOT-ICU studies,^112^^,^^113^ showed non-inferior results with potential reductions in oxygen-related adverse events.

The collective findings support a U-shaped curve relating SpO_2_ and patient outcomes, indicating that both hypoxemia and hyperoxia can be detrimental. Aiming for an SpO_2_ range of 92%–97% offers a practical and safe target, optimizing oxygen delivery while avoiding unnecessary harm. Unless otherwise specified, this SpO_2_ range (92%–97%) should be considered the default oxygenation target for most adult surgical patients throughout the intraoperative and early postoperative periods.

Exceptions may include patients with chronic hypercapnic respiratory failure, severe pulmonary hypertension, cyanotic congenital heart disease, or other conditions in which individualized oxygenation targets are clinically justified.

Oxygen therapy in surgical patients should be titrated based on real time monitoring, individual comorbidities, and surgical risk, avoiding the routine use of high FiO_2_ in patients with normal oxygenation. In parallel, ventilatory adjustments should aim to maintain arterial carbon dioxide tension (PaCO_2_) and arterial pH within physiologically acceptable ranges. In most patients, this corresponds to PaCO_2_ values close to normal and an arterial pH ≥ 7.25. Mild to moderate hypercapnia may be tolerated as part of a lung protective strategy when it represents a physiological consequence of limiting tidal volume and airway pressures to reduce lung stress, rather than inadequate ventilation, provided that arterial pH remains acceptable and that no contraindications are present, such as severe pulmonary hypertension, intracranial hypertension, or significant right ventricular dysfunction. Ventilatory settings should therefore be individualized, balancing lung protection with adequate carbon dioxide clearance and overall physiological tolerance. In addition to ventilator adjustments, supportive respiratory care measures should be maintained throughout the perioperative period. Adequate analgesia, airway humidification when indicated, endotracheal suctioning when clinically necessary, and respiratory physiotherapy according to institutional protocols may contribute to secretion clearance, patient comfort, preservation of lung function, and optimization of perioperative respiratory support.

**Table:** **Summary Table** Oxygen saturation targets and clinical implications in surgical patients.

**SpO_2_ Range**	**Clinical Implications**	**Notes**
92%–97%	Optimal range balancing oxygen delivery and avoidance of oxidative stress	Recommended target for most intra- and postoperative scenarios
> 97%	Risk of atelectasis, oxidative injury, and potential increase in mortality	Should be avoided unless clinically indicated
< 90%	Associated with increased mortality, ischemia, and adverse neurological outcomes	Requires prompt intervention to restore adequate oxygenation
Overall	Gas exchange must be interpreted globally	Oxygenation targets should be considered together with PaCO_2_ and arterial pH, ensuring physiological tolerance

SpO_2_, Peripheral Oxygen saturation.

### Cardiopulmonary interactions during intraoperative mechanical ventilation

Mechanical ventilation alters the natural cardiopulmonary physiology by applying positive airway pressure, which changes intrathoracic pressure and pulmonary volume dynamics.^94^ These effects influence venous return, cardiac preload and afterload, particularly affecting the right ventricle, and vary according to lung compliance and ventilatory settings.^55^ Understanding these interactions is crucial for optimizing hemodynamic stability and guiding protective ventilation strategies during surgery.

#### PICO clinical question

In adult surgical patients under general anesthesia and mechanical ventilation (P), how do different ventilatory modes and parameters (I), compared to conventional ventilatory strategies (C), affect cardiopulmonary interactions and hemodynamic function (O)?

**Table:** Evidence-Based Recommendations.

**Recommendation**	**GRADE Recommendation**	**Evidence Quality**
Pressure-Controlled Ventilation (PCV) and Volume-Controlled Ventilation (VCV) have similar hemodynamic effects when airway pressures are matched.	Strong	Moderate.^114^^,^^115^
Consider PC-IRV instead of VCV to improve oxygenation and reduce peak inspiratory pressure in selected patients, acknowledging potential reduction in cardiac output.	Weak	Low.^116^^,^^117^
Consider PC-IRV over PCV in ARDS patients to enhance oxygenation and CO_2_ clearance, acknowledging the potential for reduced cardiac output.	Weak	Low.^118^

PCV, Pressure-Controlled Ventilation; VCV, Volume-Controlled Ventilation; PC-IRV, Pressure-Controlled Inverse Ratio Ventilation; ARDS, Acute Respiratory Distress Syndrome; CO_2_, Carbon Dioxide; GRADE, Grading of Recommendations, Assessment, Development and Evaluation.

#### Rationale and literature-based discussion

During positive pressure ventilation, increased Intrathoracic Pressure (ITP) reduces the gradient for venous return by increasing right atrial pressure, which may reduce cardiac output.^115^ Right ventricular preload is influenced by ITP and lung volume, while right ventricular afterload is primarily determined by Pulmonary Vascular Resistance (PVR), which follows a U-shaped relationship with lung volumes being elevated both in atelectasis and overdistension.

Thus, the choice of ventilatory mode must balance the respiratory benefits with potential cardiovascular effects, especially in patients with compromised hemodynamics or preexisting heart disease.

**Table:** **Summary Table** Hemodynamic considerations for ventilatory modes.

**Mode / Strategy**	**Hemodynamic Considerations**	**Clinical Notes**
PCV vs. VCV	Equivalent cardiac output if airway pressures and tidal volumes are matched	PCV may limit peak pressures; choose based on lung mechanics and surgical needs
PC-IRV vs. VCV	May reduce cardiac output due to high mean airway pressure and decreased venous return	Potential benefit in gas exchange; use cautiously in unstable patients
PC-IRV in ARDS	Improves oxygenation and CO_2_ clearance; ↓ CO may occur without RV failure	Monitor perfusion and volume status; use individualized targets

PCV, Pressure-Controlled Ventilation; VCV, Volume-Controlled Ventilation; PC-IRV, Pressure-Controlled Inverse Ratio Ventilation; ARDS, Acute Respiratory Distress Syndrome; CO_2_, Carbon dioxide; CO, Cardiac Output; RV, Right Ventricle.

### Mechanical ventilation in the intraoperative period for patients with obstructive lung disease

Patients with obstructive lung diseases such as asthma and Chronic Obstructive Pulmonary Disease (COPD) present unique intraoperative challenges due to increased airway resistance, air trapping, dynamic hyperinflation, and risk of barotrauma.^119^ Anesthesiologists must adapt ventilation strategies to avoid complications related to positive pressure ventilation, especially when surgical factors (e.g., pneumoperitoneum, Trendelenburg position) further compromise respiratory mechanics.

#### PICO clinical question

In adult surgical patients with obstructive lung disease undergoing general anesthesia and mechanical ventilation (P), how do individualized ventilatory strategies (I), compared to standard ventilation protocols (C), affect the incidence of intraoperative and postoperative respiratory complications (O)?

**Table:** Evidence-Based Recommendations.

**Recommendation**	**GRADE Recommendation**	**Evidence Quality**
Use low tidal volumes (6–8 mL.kg^-1^ PBW) and prolonged expiratory times to minimize dynamic hyperinflation.	Strong	Moderate.^120^^,^^121^
Maintain low respiratory rates (e.g., 8–10 breaths/min) to increase expiratory time and reduce air trapping.	Conditional	Low.^122^
Monitor for auto-PEEP and consider external PEEP ≤ 80% of measured auto-PEEP only when patient triggering is impaired.	Conditional	Low.^123^, ^124^, ^125^
Avoid high PEEP and recruitment maneuvers unless clear evidence of atelectasis-induced hypoxemia.	Strong	Low.^102^
Use Pressure-Controlled Ventilation (PCV) or Volume-Controlled Ventilation (VCV) with decelerating flow pattern for optimal control of peak pressure.	Conditional	Low.^126^^,^^127^
Adjust Inspiratory-to-Expiratory (I:E) ratio (e.g., 1:3 to 1:4) and increasing inspiratory flow to minimize air trapping.	Strong	Moderate.^122^^,^^125^
Evaluate plateau pressure and driving pressure to detect early signs of dynamic hyperinflation.	Conditional	Low.^97^^,^^128^

PBW, Predicted Body Weight. For practical bedside application, the predicted body weight calculation formula and a simplified height-based reference table are provided in the Supplementary Material; auto-PEEP, Intrinsic Positive End-Expiratory Pressure; PEEP, Positive End-Expiratory Pressure; PCV, Pressure-Controlled Ventilation; VCV, Volume-Controlled Ventilation; I:E, Inspiratory-to-Expiratory ratio; GRADE, Grading of Recommendations, Assessment, Development and Evaluation.

#### Rationale and literature-based discussion

Obstructive lung diseases are characterized by increased airway resistance and expiratory flow limitation, often leading to air trapping and dynamic hyperinflation.^119^ During general anesthesia and mechanical ventilation, these pathophysiological alterations can result in increased intrathoracic pressure, auto-PEEP, impaired venous return, and hemodynamic instability.

**Table:** **Summary Table** Clinical considerations for ventilation in obstructive disease.

**Strategy**	**Clinical Consideration**
Low tidal volume (6–8 mL.kg^-1^ PBW)	Reduces barotrauma and minimizes hyperinflation
Reduced RR (e.g., 8–10 bpm)	Allows for adequate expiration and prevents air trapping
Adjusted I:E ratio (e.g., 1:3–1:4)	Enhances alveolar emptying in expiratory-limited lungs
Monitor plateau and driving pressure	Identifies risk of hyperinflation and optimizes protective ventilation
Use external PEEP only if auto-PEEP detected	Apply ≤ 80% of auto-PEEP and avoid unnecessary overdistension
Avoid routine recruitment maneuvers	May cause overdistension and hemodynamic instability in obstructive physiology

PBW, Predicted Body Weight. For practical bedside application, the predicted body weight calculation formula and a simplified height-based reference table are provided in the Supplementary Material; RR, Respiratory Rate; I:E, Inspiratory-to-Expiratory Ratio; PEEP, Positive End-Expiratory Pressure; auto-PEEP, Intrinsic Positive End-Expiratory Pressure.

### Mechanical ventilation in obese surgical patients

Obesity is an increasingly prevalent condition and presents significant challenges for perioperative management, especially regarding mechanical ventilation.^30^ Obese patients exhibit altered respiratory mechanics due to increased intra-abdominal pressure, reduced chest wall compliance, decreased Functional Residual Capacity (FRC), and a tendency for atelectasis formation. These changes increase the risk of intraoperative hypoxemia and Postoperative Pulmonary Complications (PPCs).^102^ Therefore, individualized ventilatory strategies are required to optimize gas exchange, ensure lung protection, and improve outcomes.

#### PICO clinical question

In obese adult surgical patients under general anesthesia (P), do individualized protective ventilatory strategies (I), compared to conventional ventilation (C), improve intraoperative gas exchange and reduce postoperative pulmonary complications (O)?

**Table:** Evidence-Based Recommendations.

**Recommendation**	**GRADE Recommendation**	**Evidence Quality**
Use tidal volumes between 6–8 mL.kg^-1^ of Predicted Body Weight (PBW), based on the ARDSNet formula, regardless of actual body weight.	Strong	High.^54^^,^^102^
Consider Recruitment Maneuvers (RM) in cases of persistent SpO₂ < 94%.	Weak	Low.^129^^,^^130^
Consider performing RM during PEEP titration to identify the PEEP associated with better compliance or lower driving pressure.	Conditional	Low.^129^^,^^130^
Prefer ventilator-guided RM over manual “bag-squeeze” techniques.	Strong	Moderate.^102^^,^^131^
Use a reduced FiO_2_ (e.g., 0.4–0.6) during RM to minimize reabsorption atelectasis and oxidative stress.	Strong	Moderate.^132^^,^^133^
In morbidly obese patients, plateau pressures up to 40 cm H_2_O may be required during RM, provided that close monitoring is maintained.	Conditional	Moderate.^34^^,^^129^^,^^130^
Prefer Pressure-Controlled Ventilation with Volume Guarantee (PCV-VG) when available, especially in laparoscopic procedures.	Conditional	Moderate.^48^^,^^49^
Consider conventional pressure-controlled ventilation when PCV-VG is unavailable, acknowledging potential tidal volume variability.	Weak	Low.^48^
Volume-controlled ventilation is also acceptable when PCV-VG is unavailable and fixed tidal volume delivery is a priority.	Weak	Low^49^
Maintain peak airway pressure (Ppeak) ≤ 35 lt; 35 cm H_2_O when possible.	Strong	High.^134^
Maintain plateau pressure (Pplat) ≤ 30 cm H_2_O when possible.	Strong	High.^61^^,^^135^
In specialized centers, consider using estimated transpulmonary pressure (via esophageal balloon) to guide fine-tuning of ventilation in superobese patients (BMI > 50 kg.m^-2^).	Conditional	Low.^95^^,^^136^
Aim to reduce driving pressure (ΔP) and maintain values ≤ 15 lt; 15 cm H_2_O.	Strong	Low.^64^^,^^97^^,^^128^
Maintain PEEP ≥ 5 cm H_2_O or individualized titration to optimize PaO_2_/FiO_2_ ratio intraoperatively.	Strong	Moderate.^137^^,^^138^
Consider titrating PEEP to ≥ 10 cm H_2_O to optimize PaO_2_/FiO_2_ ratio and compliance.	Strong	Low.^137^^,^^138^
Avoid low PEEP levels (< 5 lt; 5 cm H_2_O) in obese patients; individualized PEEP is safe when paired with adequate hemodynamic monitoring.	Strong	High.^102^

PBW, Predicted Body Weight. For practical bedside application, the predicted body weight calculation formula and a simplified height-based reference table are provided in the Supplementary Material; ARDSNet, Acute Respiratory Distress Syndrome Network; RM, Recruitment Maneuver; PEEP Positive End-Expiratory Pressure; FiO_2_, Fraction of inspired oxygen; PCV-VG, Pressure-Controlled Ventilation with Volume Guarantee; Ppeak, Peak inspiratory pressure; Pplat, Plateau Pressure; BMI, Body Mass Index; ΔP, Driving Pressure; PaO_2_/FiO_2_, Ratio of arterial oxygen tension to fraction of inspired oxygen; GRADE, Grading of Recommendations, Assessment, Development and Evaluation.

#### Rationale and literature-based discussion

Obesity alters respiratory mechanics by increasing pleural pressures, reducing lung volumes, and promoting airway closure. These changes reduce FRC below closing capacity and increase the risk of atelectasis, especially in the dependent lung regions.^138^ During anesthesia and mechanical ventilation, these effects are exacerbated, demanding protective ventilatory strategies.^137^

The use of tidal volumes based on Predicted Body Weight (PBW) prevents volutrauma. Even in obese patients, using actual body weight can result in excessive volumes and lung stress.^138^

**Table:** **Summary Table** Protective strategies for mechanical ventilation in obese surgical patients.

**Strategy**	**Recommended Target**	**Clinical Considerations**
Tidal Volume	6–8 mL.kg^-1^ of predicted body weight (PBW)	Prevents volutrauma; actual body weight leads to excessive VT and lung stress.
Peak Inspiratory Pressure (Ppeak)	< 35 lt; 35 cm H_2_O	Higher values may reflect increased resistance; requires monitoring of delivered VT.
Plateau Pressure (Pplat)	≤ 30 cm H_2_O	Limits alveolar overdistension, especially important in reduced compliance.
Driving Pressure (ΔP)	≤ 15 cm H_2_O	Marker of lung stress; reduction correlates with improved outcomes.
PEEP	≥ 5 cm H_2_O or individualized	Counteracts atelectasis; titration based on compliance/ΔP improves oxygenation.
Recruitment Maneuvers (RM)	Apply in hypoxemia or during PEEP titration	Ventilator-guided maneuvers preferred safety and consistency.
FiO₂ during RM	0.4–0.6	Reduces risk of absorption of atelectasis and oxygen toxicity.
Ventilatory Mode	Prefer PCV-VG; PCV or VCV acceptable	PCV-VG ensures target VT with lower peak pressures; alternatives require close monitoring.
Transpulmonary Pressure Monitoring	Consider in superobese (BMI > 50 gt; 50 kg.m^-^²)	Esophageal balloon helps individualize PEEP and limit overdistension.
Extubation	Early when feasible	Prevents prolonged exposure to high pressures; ensure adequate reversal of NMB and respiratory drive.

PBW, Predicted Body Weight. For practical bedside application, the predicted body weight calculation formula and a simplified height-based reference table are provided in the Supplementary Material; VT, Tidal Volume; Ppeak, Peak inspiratory pressure; Pplat, Plateau Pressure; ΔP, Driving Pressure; PEEP, Positive End-Expiratory Pressure; RM, Recruitment Maneuver; FiO_2_, Fraction of inspired oxygen; PCV-VG, Pressure-Controlled Ventilation with Volume Guarantee; PCV, Pressure-Controlled Ventilation; VCV, Volume-Controlled Ventilation; BMI, Body Mass Index; NMB, Neuromuscular Blockade.

### Mechanical ventilation during laparoscopic surgery

Laparoscopic surgery significantly alters respiratory mechanics through pneumoperitoneum and surgical positioning (e.g., Trendelenburg), leading to cephalad diaphragm displacement, decreased pulmonary compliance, ventilation-perfusion mismatch, and atelectasis.^139^ These changes increase the risk of PPCs, underscoring the need for lung-protective and individualized ventilation strategies.

#### Robot-assisted laparoscopic surgery

Robot-assisted laparoscopic procedures deserve specific consideration within the laparoscopic context, because they frequently involve steeper and more sustained Trendelenburg positioning, longer operative times, and limited physical access to the patient after docking. These factors can accentuate reductions in respiratory system compliance, increase airway pressures, and make frequent circuit adjustments or recruitment maneuvers less practical once docking is complete.^139^ In this setting, protective ventilation based on predicted body weight remains the default strategy, but it becomes particularly important to anticipate physiologic deterioration, to monitor respiratory mechanics trends closely, and to prioritize ventilatory adjustments that can be implemented safely without repeated disconnections, such as careful titration of PEEP, reassessment of driving pressure, and attention to hemodynamic tolerance during any recruitment strategy.^140^ This pragmatic framing aligns the general principles of laparoscopy with the operational constraints and physiologic burden commonly seen during robotic surgery in steep Trendelenburg.

#### PICO clinical question

In adult surgical patients undergoing laparoscopic procedures (P), do tailored ventilatory adjustments (I), compared to conventional strategies (C), improve oxygenation and reduce postoperative pulmonary complications (O)?

**Table:** Evidence-Based Recommendations.

**Recommendation**	**GRADE Recommendation**	**Evidence Quality**
Use protective ventilation (6–8 mL.kg^-1^ predicted body weight + PEEP ≈5 cm H_2_O) as the standard intraoperative strategy.	Strong	High.^141^^,^^142^
Avoid conventional ventilation (8–10 mL.kg^-1^, no PEEP) due to increased complications and poorer oxygenation.	Strong	High.^141^^,^^143^
Consider using PCV-VG over VCV to reduce airway pressures and improve compliance.	Weak	Low.^144^^,^^145^
Consider using PCV instead of VCV for improved inflammatory response and compliance in select patients.	Weak	Low.^143^^,^^146^
Prefer individualized PEEP titration (guided by ΔP, compliance, or EIT) rather than fixed PEEP (e.g., 5 cm H_2_O or ZEEP).	Strong	High.^147^^,^^148^
Consider alveolar Recruitment Maneuvers (RMs) to restore compliance and reduce atelectasis, especially after pneumoperitoneum deflation.	Weak	Moderate.^149^^,^^150^

PBW, Predicted Body Weight. For practical bedside application, the predicted body weight calculation formula and a simplified height-based reference table are provided in the Supplementary Material; PEEP, Positive End-Expiratory Pressure; PCV-VG, Pressure-Controlled Ventilation with Volume Guarantee; VCV, Volume-Controlled Ventilation; PCV, Pressure-Controlled Ventilation; ΔP, Driving Pressure; EIT, Electrical Impedance Tomography; ZEEP, Zero End-Expiratory Pressure; RM, Recruitment Maneuver; GRADE, Grading of Recommendations, Assessment, Development and Evaluation.

#### Rationale and literature-based discussion

Laparoscopic surgery induces physiological alterations that reduce functional residual capacity, displace the diaphragm upward, and promote lung collapse, especially in dependent regions. Protective ventilation strategies have been associated with decreased incidence of atelectasis, hypoxemia, and VILI.^142^ Conventional ventilation increases pulmonary stress and systemic inflammation, even in patients with healthy lungs, and should be avoided.^137^

**Table:** **Summary Table** Ventilatory strategies in laparoscopic surgery.

**Strategy**	**Impact / Recommendation**
Protective ventilation (6–8 mL.kg^-1^ + PEEP)	Reduces PPCs and improves oxygenation
Avoid conventional ventilation (no PEEP)	Increases risk of VILI and complications
PCV-VG over VCV	Reduces peak pressures and improves ΔP
PCV over VCV	May reduce airway pressures
Individualized PEEP	Improves compliance and intraoperative oxygenation
Recruitment maneuvers (after desufflation)	Restore compliance and reduce atelectasis

PEEP, Positive End-Expiratory Pressure; PPCs, Postoperative Pulmonary Complications; VILI, Ventilator-Induced Lung Injury; PCV-VG, Pressure-Controlled Ventilation with Volume Guarantee; VCV, Volume-Controlled Ventilation; PCV, Pressure-Controlled Ventilation; ΔP, Driving Pressure.

#### Recommendation in the area of clinical uncertainty

Pressure-Controlled Ventilation (PCV) compared with Volume-Controlled Ventilation (VCV) did not reach the predefined threshold of expert consensus in the Delphi process. While PCV may confer physiological advantages, such as reduced peak inspiratory pressures in patients with impaired pulmonary compliance, the current body of evidence is insufficient and inconsistent with respect to meaningful clinical outcomes. This intervention should therefore be regarded as falling within an area of clinical uncertainty, highlighting the need for further high-quality randomized controlled trials before routine adoption into perioperative practice can be recommended.

### Mechanical ventilation in critically Ill neurosurgical patients

**Figure 3:**
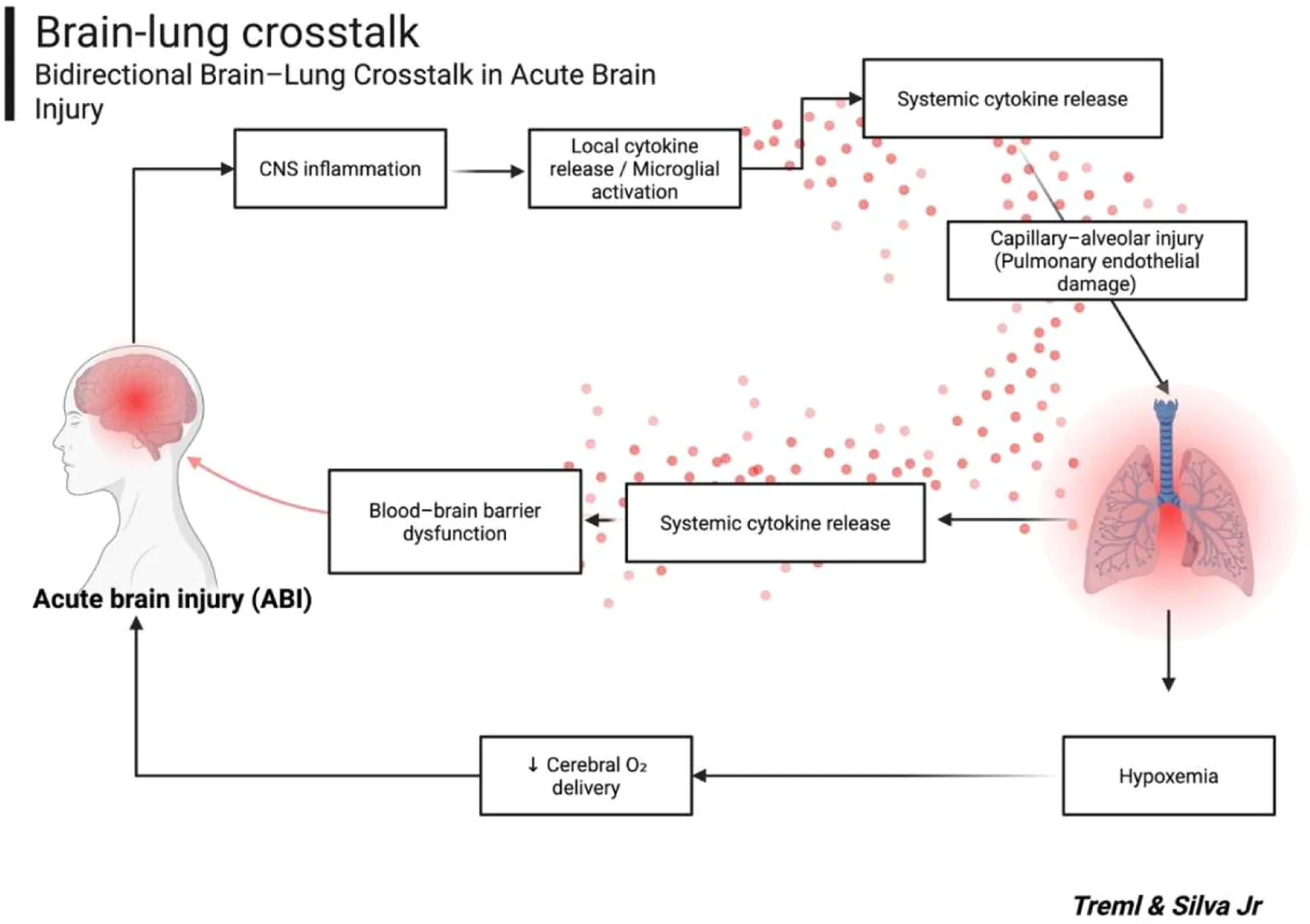
Brain-lung crosstalk. ABI, Acute Brain Injury; O_2_, Oxygen; CNS, Central Nervous System.

Mechanical ventilation in critically ill neurosurgical patients presents unique challenges. It requires balancing optimal gas exchange and protective ventilation while avoiding secondary neurological insults. Elevated Intracranial Pressure (ICP), impaired autoregulation, and altered cerebrovascular reactivity must all be considered when defining ventilation strategies during the perioperative period (Fig. 3).^151^

#### PICO clinical question

In adult neurosurgical patients under general anesthesia (P), do protective ventilatory strategies (I), compared to conventional strategies (C), optimize gas exchange while maintaining intracranial and cerebral hemodynamic stability (O)?

**Table:** Evidence-Based Recommendations.

**Recommendation**	**GRADE Recommendation**	**Evidence Quality**
Protective ventilation (VT 6–8 mL.kg^-1^ PBW + PEEP ≈5 cm H_2_O) should be the standard approach in neurosurgical patients.	Strong	High.^151^^,^^152^
Avoid high tidal volumes (> 10 gt; 10 mL.kg^-1^ PBW) and ZEEP ventilation.	Strong	High.^152^^,^^153^
Avoid permissive hypercapnia (PaCO_2_ > 45 mmHg) in patients with elevated ICP or impaired autoregulation.	Strong	Moderate.^154^^,^^155^
Maintain normocapnia (PaCO_2_ 35–40 mmHg), especially in patients with elevated ICP.	Strong	Moderate.^154^^,^^155^
In patients with invasive neuromonitoring (ICP, PbtO_2_), consider individualized CO_2_ targets guided by cerebral parameters.	Conditional	Low.^156^^,^^155^

VT, Tidal Volume; PBW, Predicted Body Weight. For practical bedside application, the predicted body weight calculation formula and a simplified height-based reference table are provided in the Supplementary Material; PEEP, Positive End-Expiratory Pressure; ZEEP, Zero End-Expiratory Pressure; PaCO_2_, Arterial Carbon dioxide tension; ICP, Intracranial Pressure; PbtO_2_, Brain tissue oxygen tension; GRADE, Grading of Recommendations, Assessment, Development and Evaluation.

#### Rationale and literature-based discussion

Ventilatory management in acute brain injury requires balancing cerebral perfusion and Intracranial Pressure (ICP), both of which are highly sensitive to arterial CO₂. While hypercapnia increases cerebral blood flow and ICP, hypocapnia can reduce ICP but risks cerebral hypoperfusion, especially in patients with impaired autoregulation. For this reason, maintaining normocapnia (PaCO_2_ 35–40 mmHg) is generally recommended,^155^ and routine hypocapnia should be avoided due to its association with increased mortality.^154^ Protective ventilation with moderate tidal volumes (6–8 mL.kg^-1^ PBW) and low PEEP (∼5 cm H_2_O) is generally safe and minimizes lung injury without worsening ICP, as long as perfusion is preserved.^156^ However, the PROLABI trial showed increased mortality in patients without ARDS using this strategy, suggesting it should not be applied universally.^153^

Avoiding high tidal volumes and ZEEP is critical, as both are linked to pulmonary complications and systemic inflammation, which may worsen neurological outcomes.^152^

When neuromonitoring is available, CO_2_ targets and ventilator settings should be individualized. Although hypercapnia may be tolerated in ARDS, it is contraindicated in brain injury due to the risk of exacerbating ICP and cerebral edema.^155^

**Table:** **Summary Table** Key ventilation strategies in surgical patients with acute brain injury.

**Clinical Strategy**	**Practical Considerations and Physiological Impact**
Protective ventilation (VT 6–8 mL.kg^-1^ PBW + PEEP ≈5)	Minimizes VILI and atelectasis; well tolerated in most patients; monitor closely in absence of ARDS
Avoid high VT (10 mL.kg^-1^) and ZEEP	Associated with increased risk of atelectasis, pneumonia, inflammation, and neuroinjury biomarkers.
Moderate PEEP ≥ 5 cm H_2_O	Supports alveolar recruitment; safe for cerebral hemodynamics in stable patients.
Maintain normocapnia (PaCO_2_ 35–40 mmHg)	Optimizes balance between cerebral perfusion and ICP; preferred in most scenarios.
Avoid permissive hypercapnia	May worsen ICP and cerebral edema; not recommended unless under advanced neuromonitoring.
Individualized CO_2_ targets	Adjust PaCO_2_ based on ICP, PbtO_2_, or jugular venous saturation when monitoring is available.

VT, Tidal Volume; PBW, Predicted Body Weight. For practical bedside application, the predicted body weight calculation formula and a simplified height-based reference table are provided in the Supplementary Material; PEEP, Positive End-Expiratory Pressure; VILI, Ventilator-Induced Lung Injury; ICP, Intracranial Pressure; PaCO_2_, Arterial Carbon Dioxide Tension; PbtO_2_, Brain tissue oxygen tension.

### Mechanical ventilation in thoracic surgery

Thoracic surgery poses unique challenges for intraoperative ventilation. One-Lung Ventilation (OLV), often required for exposure, alters mechanics, reduces functional residual capacity, and increases the risk of hypoxemia and VILI. Protective strategies are therefore essential to ensure gas exchange and reduce complications.^157^

Given the high incidence of hypoxemia during one-lung ventilation, a structured stepwise approach is essential to guide timely corrective interventions. A practical one-page algorithm for the management of hypoxemia during OLV is provided in the Supplementary Material (Supplementary Appendix S3).

#### PICO clinical question

In adult patients undergoing thoracic surgery requiring one-lung ventilation (P), do protective ventilatory strategies (I), compared to conventional ventilation (C), improve oxygenation and reduce postoperative pulmonary complications (O)?

**Table:** Evidence-Based Recommendations.

**Recommendation**	**GRADE Recommendation**	**Evidence Quality**
Use low tidal volumes (4–6 mL.kg^-1^ PBW) during OLV to reduce lung stress and prevent VILI.	Strong	High.^158^^,^^159^
Apply moderate PEEP (5–10 cm H_2_O) to the dependent lung to prevent atelectasis during OLV.	Strong	Moderate.^160^^,^^161^
Avoid routine use of ZEEP during OLV due to increased risk of alveolar collapse.	Strong	Moderate.^162^^,^^163^
Prefer Pressure-Controlled Ventilation (PCV) or volume-guaranteed pressure modes to limit peak pressure.	Conditional	Moderate.^47^^,^^164^
Maintain plateau pressure ≤ 30 lt; 30 cm H_2_O and driving pressure ideally ≤ 12 lt; 12 cm H_2_O, maximum 15 cm H_2_O.	Strong	Moderate.^165^^,^^166^
Consider individualized PEEP titration based on driving pressure or compliance.	Conditional	Low.^167^
Avoid unnecessary hyperoxia; target SpO_2_ 92%–96% during OLV.	Strong	Moderate.^168^^,^^169^
Recruitment maneuvers may be considered as rescue therapy for severe hypoxemia.	Weak	Low.^163^^,^^170^

PBW, Predicted Body Weight. For practical bedside application, the predicted body weight calculation formula and a simplified height-based reference table are provided in the Supplementary Material; OLV, One-Lung Ventilation; VILI, Ventilator-Induced Lung Injury; PEEP, Positive End-Expiratory Pressure; ZEEP, Zero End-Expiratory Pressure; PCV, Pressure-Controlled Ventilation; FiO_2_, Fraction of inspired oxygen; SpO_2_, Peripheral Oxygen saturation; GRADE, Grading of Recommendations, Assessment, Development and Evaluation.

#### Rationale and literature-based discussion

Thoracic surgery with OLV imposes unique challenges, including increased risk of hypoxemia, volutrauma, and atelectrauma due to asymmetric ventilation. Protective ventilation strategies are critical to mitigate these risks.^162^

During OLV, hypoxemia is predominantly driven by shunt and ventilation–perfusion mismatch and may be exacerbated by tube malposition, inadequate PEEP in the dependent lung, or excessive collapse of the non-dependent lung. A systematic evaluation of these factors is therefore required before escalation of rescue therapies.

RCTs and meta-analyses confirm that low tidal volumes (4–6 mL.kg^-1^ PBW) reduce barotrauma and Postoperative Pulmonary Complications (PPCs) during OLV.^157^ Recruitment maneuvers during OLV should not be applied routinely and are best reserved as rescue interventions for severe or refractory hypoxemia, with careful attention to hemodynamic tolerance and surgical conditions.

In cases of refractory hypoxemia during one-lung ventilation, additional rescue strategies may be considered after optimization of ventilatory settings and oxygenation measures. These include selective pulmonary vasodilators, jet ventilation to the non-dependent lung, and optimization of anesthetic depth to preserve hypoxic pulmonary vasoconstriction when clinically appropriate. Although evidence supporting these interventions remains limited, they may be useful in selected patients and should be individualized according to surgical, physiological, and hemodynamic conditions. A practical stepwise approach to the management of hypoxemia during one-lung ventilation is provided in Supplementary Appendix S3.

**Table:** **Summary Table** Protective ventilation strategies for thoracic surgery.

**Strategy**	**Clinical Application and Considerations**
Low tidal volume (4–6 mL.kg^-1^ PBW)	Reduces alveolar overdistension and risk of VILI during OLV
Moderate PEEP (5–10 cm H_2_O)	Improves oxygenation and lung compliance in the dependent lung
Plateau pressure ≤ 30 cm H_2_O	Avoids excessive alveolar stretch and barotrauma
Driving Pressure ≤ 15 lt; 15 cm H_2_O (ideally ≤ 12 lt; 12 cm H_2_O)	Strong predictor of outcomes.
Pressure-controlled or PCV-VG modes	May reduce peak inspiratory pressure and improve distribution of ventilation
Avoid hyperoxia	Target SpO_2_ between 92%–96% to prevent oxidative injury
Individualized PEEP titration	Optimize compliance and driving pressure in selected patients
Recruitment Rescue in severe hypoxemia	Use cautiously, not routine.

PBW, Predicted Body Weight. For practical bedside application, the predicted body weight calculation formula and a simplified height-based reference table are provided in the Supplementary Material; VILI, Ventilator-Induced Lung Injury; OLV, One-Lung Ventilation; PEEP, Positive End-Expiratory Pressure; PCV, Pressure-Controlled Ventilation; PCV-VG, Pressure-Controlled Ventilation with Volume Guarantee; SpO_2_, Peripheral Oxygen saturation.

#### Ventilatory strategies during cardiac surgery

Cardiac surgery with Cardiopulmonary Bypass (CPB) induces significant pulmonary dysfunction due to factors like lung deflation, ischemia-reperfusion, hypothermia, and systemic inflammation, leading to atelectasis, impaired gas exchange, and increased risk of PPCs.^171^

#### PICO clinical question

In adult patients undergoing cardiac surgery with cardiopulmonary bypass (P), do lung-protective ventilation strategies, including recruitment maneuvers and individualized PEEP (I), compared to conventional ventilation (C), improve gas exchange and reduce postoperative pulmonary complications (O)?

**Table:** Evidence-Based Recommendations.

**Recommendation**	**GRADE Recommendation**	**Evidence Quality**
Perform alveolar recruitment maneuvers at the end of CPB to re-expand collapsed lung regions.	Strong	Moderate.^172^, ^173^, ^174^
Use individualized PEEP titration (e.g., based on compliance or driving pressure) after RM to maintain alveolar patency.	Conditional	Moderate.^173^^,^^174^
Avoid high fixed PEEP levels without prior RM due to potential overdistension and hemodynamic compromise.	Strong	Moderate.^171^^,^^172^
Use protective ventilation (6–8 mL.kg^-1^ PBW, Pplat ≤ 30 cm H_2_O) throughout surgery, including before and after CPB.	Strong	Low.^10^^,^^175^

CPB, Cardiopulmonary Bypass; PEEP, Positive End-Expiratory Pressure; RM, Recruitment Maneuver; PBW, Predicted Body Weight. For practical bedside application, the predicted body weight calculation formula and a simplified height-based reference table are provided in the Supplementary Material; Pplat, Plateau Pressure; GRADE, Grading of Recommendations, Assessment, Development and Evaluation.

#### Rationale and literature-based discussion

Cardiopulmonary Bypass (CPB) leads to transient but significant loss of pulmonary aeration due to factors like lung deflation during extracorporeal circulation and systemic inflammation. Studies show that this promotes atelectasis formation, gas exchange impairment, and subsequent development of PPCs such as hypoxemia, pneumonia, or acute lung injury.^176^

During cardiopulmonary bypass, lung ventilation is often reduced or temporarily interrupted, which may further exacerbate atelectasis and pulmonary inflammation. When technically feasible, the application of low-level continuous positive airway pressure or very low tidal volume ventilation during CPB has been proposed to mitigate lung collapse, although available evidence remains heterogeneous and does not support a uniform approach. Therefore, ventilatory management during CPB should be individualized according to surgical conditions and institutional practice, with emphasis on prompt re-establishment of lung-protective ventilation strategies immediately after separation from bypass.

Recruitment Maneuvers (RMs) following CPB have been shown to reopen collapsed alveolar units and restore functional residual capacity. When followed by PEEP titration based on best compliance or driving pressure, the likelihood of alveolar stability increases, and oxygenation is maintained.^172^

**Table:** **Summary Table** Protective strategies in cardiac surgery.

**Strategy**	**Clinical Application and Considerations**
Alveolar recruitment maneuvers post-CPB	Re-expand atelectatic lung regions and restore aeration
Individualized PEEP titration	Maintains alveolar patency and optimizes compliance or driving pressure
Avoid high fixed PEEP without RM	Prevents overdistension and hemodynamic instability
Protective ventilation (6–8 mL.kg^-1^ PBW)	Minimizes VILI and supports lung-protective intraoperative care

CPB, Cardiopulmonary Bypass; PEEP, Positive End-Expiratory Pressure; RM, Recruitment Maneuver; PBW, Predicted Body Weight. For practical bedside application, the predicted body weight calculation formula and a simplified height-based reference table are provided in the Supplementary Material; VILI, Ventilator-Induced Lung Injury.

### Pediatric intraoperative mechanical ventilation: modalities and settings

Perioperative mechanical ventilation in pediatric patients can negatively impact pulmonary function even in the absence of pre-existing lung disease.^177^ Specific ventilatory strategies can minimize complications such as atelectasis, hypoxemia, and VILI, promoting better perioperative outcomes.^178^ Evidence in pediatric perioperative ventilation remains limited, with most recommendations extrapolated from adult studies or based on small pediatric trials.^179^ Therefore, these recommendations should be interpreted with caution and adapted to individual patient characteristics.

#### PICO clinical question

In pediatric surgical patients under general anesthesia (P), do protective ventilatory strategies (I), compared to conventional ventilation (C), improve intraoperative oxygenation and reduce postoperative pulmonary complications (O)?

**Table:** Evidence-Based Recommendations.

**Recommendation**	**GRADE Recommendation**	**Evidence Quality**
Use Pressure-Controlled Ventilation with Volume Guarantee (PCV-VG) in healthy pediatric patients.	Weak	Moderate.^180^
Consider Pressure Support Ventilation (PSV) with PEEP to maintain spontaneous breathing.	Conditional	Moderate.^181^^,^^182^
Avoid spontaneous ventilation without ventilatory support.	Strong	Moderate.^182^
Use tidal volumes of 5–8 mL.kg^-1^ of ideal body weight.	Strong	High.^177^^,^^179^
Avoid tidal volumes > 10 gt; 10 mL.kg^-1^.	Strong	High.^177^^,^^179^
Plateau pressure ≤ 28 cm H_2_O in healthy lungs or ≤ 32 cm H_2_O in obstructive disease.	Conditional	Moderate.^183^^,^^184^
Maintain driving pressure ≤ 15 lt; 15 cm H_2_O.	Conditional	Moderate.^183^
Use PEEP between 5–8 cm H_2_O to prevent alveolar collapse.	Conditional	Low.^178^
Perform intermittent alveolar recruitment maneuvers, particularly after induction or disconnection.	Strong	High.^185^^,^^186^
Combine recruitment maneuvers with PEEP.	Conditional	High.^185^^,^^186^
Use bedside lung ultrasound to guide recruitment strategies and detect atelectasis.	Conditional	Moderate.^187^
Avoid routine use of FiO_2_ = 1.0 due to risk of absorption atelectasis and heterogeneous ventilation.	Strong	High.^188^^,^^189^
Prefer FiO_2_ < 0.8 throughout the perioperative period, titrated according to need.	Conditional	Moderate.^188^
Consider use of PEEP or CPAP during induction and emergence to reduce atelectasis.	Conditional	Low.^189^

PCV-VG, Pressure-Controlled Ventilation with Volume Guarantee; PSV, Pressure Support Ventilation; PEEP, Positive End-Expiratory Pressure; PBW, Predicted Body Weight. For practical bedside application, the predicted body weight calculation formula and a simplified height-based reference table are provided in the Supplementary Material; ΔP, Driving pressure; RM, Recruitment Maneuver; FiO_2_, Fraction of inspired oxygen; CPAP, Continuous Positive Airway Pressure; GRADE, Grading of Recommendations, Assessment, Development and Evaluation.

#### Rationale and literature-based discussion

Pediatric patients are more vulnerable to ventilator-induced lung injury because of their small airway diameter, high chest wall compliance, and immature alveoli^177^, and thus strategies that minimize airway pressures are preferred. PCV-VG achieves the target tidal volume with lower airway pressures. PCV may reduce barotrauma risk, while VCV remains safe with modern ventilators despite variations in respiratory compliance.^180^ Moreover, supporting spontaneous breathing with PSV and PEEP reduces atelectasis and facilitates recovery compared to unsupported ventilation.^181^^,^^182^ Likewise, using tidal volumes of 5–8 mL.kg^-1^ reduces inflammation, whereas higher volumes increase complications, and limiting plateau and driving pressures further prevents lung injury.^179^^,^^183^ In addition, PEEP stabilizes alveoli and prevents collapse, while recruitment maneuvers and bedside lung ultrasound help reopen and detect atelectasis.^178^ Finally, high oxygen fractions (= 1.0) promote absorption atelectasis and heterogeneity, whereas keeping FiO_2_ below 0.8 and using CPAP or PEEP during induction and emergence improves homogeneity and reduces collapse.^189^

**Table:** **Summary Table** Pediatric protective ventilation strategies.

**Strategy**	**Application in Pediatric Intraoperative Ventilation**
PCV-VG or PCV modes	Reduced barotrauma risk and better volume control
PSV + PEEP	Maintains spontaneous ventilation with reduced atelectasis risk
Avoid unsupported spontaneous breathing	Risk of hypoventilation and alveolar collapse
Tidal volume 5–8 mL.kg^-1^	Optimal lung protection
Plateau pressure ≤ 28–32 cm H_2_O	Minimizes barotrauma
Driving pressure ≤ 15 lt; 15 cm H_2_O	Limits lung stress
PEEP 5–8 cm H_2_O	Prevents atelectasis
Recruitment maneuvers	Re-expand collapsed alveoli post-induction or disconnection
Bedside lung ultrasound	Atelectasis detection and recruitment guidance
FiO_2_ < 0.8	Reduces oxidative injury and ventilation heterogeneity
PEEP/CPAP during induction/emergence	Prevents collapse and improves recovery

PCV-VG, Pressure-Controlled Ventilation with Volume Guarantee; PCV, Pressure-Controlled Ventilation; PSV, Pressure Support Ventilation; PEEP, Positive End-Expiratory Pressure; ΔP, Driving Pressure; FiO_2_, Fraction of inspired oxygen; CPAP, Continuous Positive Airway Pressure.

## Discussion

This consensus guideline represents the most comprehensive effort to date in Brazil to standardize perioperative mechanical ventilation practices based on current evidence. While protective strategies are well established in critical care, their translation into the intraoperative setting has historically been inconsistent. Our recommendations reinforce the concept that even short-term exposure to injurious ventilatory patterns, such as high tidal volumes, low or absent PEEP, excessive FiO_2_, or unchecked driving pressures may result in atelectasis, impaired oxygenation, ventilator-induced lung injury, and ultimately, PPCs.

A central strength of this work is its structured methodology: 17 clinical questions formulated using the PICO framework, systematic literature searches, evidence grading with the GRADE system, and consensus-building through panel discussions. This process ensured transparency, reproducibility, and alignment with international standards. Furthermore, the inclusion of specific surgical populations (obese, pediatric, thoracic and cardiac surgery, and patients with or without acute lung injury) increases the applicability of these recommendations to diverse perioperative contexts.

Several key principles emerged consistently across chapters:

− Protective ventilation strategies should be applied to all surgical patients, not only to those at high risk or with preexisting lung disease.^31^^,^^138^^,^^177^
− Individualization of PEEP and FiO_2_ is essential to optimize gas exchange while minimizing harm.^34^^,^^74^^,^^98^
− Driving pressure and plateau pressure are valuable bedside markers of lung protection and should be monitored routinely.^64^^,^^65^^,^^128^
− Recruitment maneuvers should not be used indiscriminately but remain an important rescue intervention in selected patients.^34^^,^^129^^,^^131^
− Standardized definitions of PPCs are crucial for clinical care, research, and benchmarking.^19^, ^20^, ^21^

### Implementation and quality indicators

The clinical impact of perioperative mechanical ventilation strategies depends not only on the strength of evidence, but also on their consistent implementation and monitoring in routine practice. Translating these recommendations into institutional protocols, checklists, and education programs may facilitate adherence and reduce unwarranted variability in perioperative care.

The use of simple and measurable quality indicators may support implementation and audit processes. Examples include the proportion of patients ventilated with tidal volumes based on predicted body weight, documentation of intraoperative oxygenation targets, avoidance of unnecessary hyperoxia, and the systematic identification of postoperative pulmonary complications using standardized definitions. Monitoring these indicators over time allows benchmarking, identification of gaps in care, and continuous quality improvement.

Importantly, implementation strategies should be adapted to local resources, case mix, and organizational structures, ensuring feasibility while preserving the core principles of lung protection and patient safety outlined in this consensus.

### Strengths and limitations

Despite these strengths, limitations should be acknowledged. The available evidence in some areas, particularly advanced monitoring, pediatric patients, and neurosurgical or cardiac surgery contexts remains limited or heterogeneous. As such, some recommendations are conditional and based on moderate or low certainty of evidence. Furthermore, the guideline reflects consensus among Brazilian experts and may require contextual adaptation in other health systems with different resources and perioperative practices. Ongoing research and periodic updates will be necessary to maintain relevance as new evidence emerges.

## Conclusion

This guideline provides an evidence-based, consensus-driven framework for perioperative mechanical ventilation in adult surgical patients. By addressing 17 structured clinical questions across diverse perioperative scenarios, it emphasizes the consistent application of protective ventilation strategies, the standardization of definitions, and the individualization of ventilatory settings to patient physiology and surgical context.

Adoption of these recommendations by anesthesiologists and perioperative teams is expected to reduce postoperative pulmonary complications, improve patient outcomes, and harmonize clinical practice across institutions in Brazil. These guidelines are also a foundation for future research and continuous improvement in perioperative respiratory care. These recommendations should be periodically reviewed and updated, ideally every five years or sooner if disruptive new evidence emerges to ensure ongoing alignment with best practices in perioperative respiratory care.

## Data availability statement

The datasets generated and/or analyzed during the current study are available from the corresponding author upon reasonable request.

## AI disclosure statement

The authors used ChatGPT (OpenAI) exclusively for language editing and text refinement. All scientific content, evidence appraisal, recommendations, interpretations, and conclusions were developed, reviewed, and approved by the authors, who take full responsibility for the manuscript.

## Funding

This work was supported by the Brazilian Society of Anesthesiology (SBA).

## Conflicts of interest

The authors declare no conflicts of interest.

## Appendix Supplementary materials

Image, application 1

## Footnotes

Supplementary material associated with this article can be found in the online version at doi:10.1016/j.bjane.2026.844814.

## References

[1] Zhang N.R., Zhang L.Z., Chen Y. (2025). Intraoperative protective ventilation with or without periodic lung recruitment manoeuvres on pulmonary complications after major abdominal surgery (REMAIN-1): protocol for a randomised controlled trial. BMJ Open.

[2] Miskovic A., Lumb AB. (2017). Postoperative pulmonary complications. Br J Anaesth.

[3] Robba C., Hemmes S.N.T., Serpa Neto A. (2020). Intraoperative ventilator settings and their association with postoperative pulmonary complications in neurosurgical patients: post-hoc analysis of LAS VEGAS study. BMC Anesthesiol.

[4] Rothen H.U., Sporre B., Engberg G., Wegenius G., Hedenstierna G. (1998). Airway closure, atelectasis and gas exchange during general anaesthesia. Br J Anaesth.

[5] Gerlach R.M., Shahul S., Wroblewski K.E. (2019). Intraoperative Use of Nondepolarizing Neuromuscular Blocking Agents During Cardiac Surgery and Postoperative Pulmonary Complications: A Prospective Randomized Trial. J Cardiothorac Vasc Anesth.

[6] Hedenstierna G. (2003). Alveolar collapse and closure of airways: regular effects of anaesthesia. Clin Physiol Funct Imaging.

[7] Hedenstierna G., Strandberg A., Brismar B., Lundquist H., Svensson L., Tokics L. (1985). Functional residual capacity, thoracoabdominal dimensions, and central blood volume during general anesthesia with muscle paralysis and mechanical ventilation. Anesthesiology.

[8] Kirmeier E., Eriksson L.I., Lewald H. (2019). Post-anaesthesia pulmonary complications after use of muscle relaxants (POPULAR): a multicentre, prospective observational study. Lancet Respir Med.

[9] Scaramuzzo G., Karbing D.S., Ball L. (2024). Intraoperative Ventilation/Perfusion Mismatch and Postoperative Pulmonary Complications after Major Noncardiac Surgery: A Prospective Cohort Study. Anesthesiology.

[10] Mathis M.R., Duggal N.M., Likosky D.S. (2019). Intraoperative Mechanical Ventilation and Postoperative Pulmonary Complications after Cardiac Surgery. Anesthesiology.

[11] Lumb A.B., Slinger P. (2015). Hypoxic pulmonary vasoconstriction: physiology and anesthetic implications. Anesthesiology.

[12] Griffiths M.J.D., McAuley D.F., Perkins G.D. (2019). Guidelines on the management of acute respiratory distress syndrome. BMJ Open Respir Res.

[13] Jaeschke R., Guyatt G.H., Dellinger P. (2008). Use of GRADE grid to reach decisions on clinical practice guidelines when consensus is elusive. BMJ.

[14] Guyatt G.H., Oxman A.D., Vist G.E. (2008). GRADE: an emerging consensus on rating quality of evidence and strength of recommendations. BMJ.

[15] Balshem H., Helfand M., Schunemann H.J. (2011). GRADE guidelines: 3. Rating the quality of evidence. J Clin Epidemiol.

[16] Atkins D., Best D., Briss P.A. (2004). Grading quality of evidence and strength of recommendations. BMJ.

[17] Ansari M.T., Tsertsvadze A., Moher D. (2009). Grading quality of evidence and strength of recommendations: a perspective. PLoS Med.

[18] Scrimgeour D.S.G., Allan M., Knight S.R. (2022). A modified Delphi process to establish research priorities in hernia surgery. Hernia.

[19] Jammer I., Wickboldt N., Sander M. (2015). Standards for definitions and use of outcome measures for clinical effectiveness research in perioperative medicine: European Perioperative Clinical Outcome (EPCO) definitions: a statement from the ESA-ESICM joint taskforce on perioperative outcome measures. Eur J Anaesthesiol.

[20] Barnes J., Hunter J., Harris S. (2019). Systematic review and consensus definitions for the Standardised Endpoints in Perioperative Medicine (StEP) initiative: infection and sepsis. Br J Anaesth.

[21] Moonesinghe S.R., Jackson A.I.R., Boney O. (2019). Systematic review and consensus definitions for the Standardised Endpoints in Perioperative Medicine initiative: patient-centred outcomes. Br J Anaesth.

[22] Horan T.C., Andrus M., Dudeck M.A. (2008). CDC/NHSN surveillance definition of health care-associated infection and criteria for specific types of infections in the acute care setting. Am J Infect Control.

[23] Matthay M.A., Arabi Y., Arroliga A.C. (2024). A New Global Definition of Acute Respiratory Distress Syndrome. Am J Respir Crit Care Med.

[24] Abbott T.E.F., Fowler A.J., Pelosi P. (2018). A systematic review and consensus definitions for standardised end-points in perioperative medicine: pulmonary complications. Br J Anaesth.

[25] Costa Leme A., Hajjar L.A., Volpe M.S. (2017). Effect of Intensive vs Moderate Alveolar Recruitment Strategies Added to Lung-Protective Ventilation on Postoperative Pulmonary Complications: A Randomized Clinical Trial. JAMA.

[26] Kroenke K., VA Lawrence, Theroux J.F., Tuley MR. (1992). Operative risk in patients with severe obstructive pulmonary disease. Arch Intern Med.

[27] Fernandez-Bustamante A., Frendl G., Sprung J. (2017). Postoperative Pulmonary Complications, Early Mortality, and Hospital Stay Following Noncardiothoracic Surgery: A Multicenter Study by the Perioperative Research Network Investigators. JAMA Surg.

[28] Fernandez-Bustamante A., Parker R.A., Sprung J. (2023). An anesthesia-centered bundle to reduce postoperative pulmonary complications: The PRIME-AIR study protocol. PLoS One.

[29] Sun Q., Zhang T., Liu J., Cui Y., Tan W. (2023). A 20-year bibliometric analysis of postoperative pulmonary complications: 2003-2022. Heliyon.

[30] Neto A.S., da Costa L.G.V., Hemmes S.N.T. (2018). The LAS VEGAS risk score for prediction of postoperative pulmonary complications: An observational study. Eur J Anaesthesiol.

[31] Ferrando C., Soro M., Unzueta C. (2018). Individualised perioperative open-lung approach versus standard protective ventilation in abdominal surgery (iPROVE): a randomised controlled trial. Lancet Respir Med.

[32] Brinson E.L., Thornton KC. (2018). Preoperative Risk Assessment of Respiratory Failure. Int Anesthesiol Clin.

[33] Lim C.H., Han J.Y., Cha S.H., Kim Y.H., Yoo K.Y., Kim HJ. (2021). Effects of high versus low inspiratory oxygen fraction on postoperative clinical outcomes in patients undergoing surgery under general anesthesia: A systematic review and meta-analysis of randomized controlled trials. J Clin Anesth.

[34] Mazzinari G., Zampieri F.G., Ball L. (2022). Effect of intraoperative PEEP with recruitment maneuvers on the occurrence of postoperative pulmonary complications during general anesthesia–protocol for Bayesian analysis of three randomized clinical trials of intraoperative ventilation. F1000Res.

[35] Canet J., Gallart L., Gomar C. (2010). Prediction of postoperative pulmonary complications in a population-based surgical cohort. Anesthesiology.

[36] Silvanus M.T., Groeben H., Peters J. (2004). Corticosteroids and inhaled salbutamol in patients with reversible airway obstruction markedly decrease the incidence of bronchospasm after tracheal intubation. Anesthesiology.

[37] Smetana G.W., Lawrence V.A., Cornell J.E. (2006). American College of P. Preoperative pulmonary risk stratification for noncardiothoracic surgery: systematic review for the American College of Physicians. Ann Intern Med.

[38] Fiddes R.A., McCaffrey N. (2024). Preoperative Smoking-Cessation Interventions to Prevent Postoperative Complications: A Quality Assessment and Overview of Systematic Review Evidence. Anesth Analg.

[39] Wong C., Mohamad Asfia S.K.B., Myles P.S. (2025). Smoking and Complications After Cancer Surgery: A Systematic Review and Meta-Analysis. JAMA Netw Open.

[40] Boden I., Skinner E.H., Browning L. (2018). Preoperative physiotherapy for the prevention of respiratory complications after upper abdominal surgery: pragmatic, double blinded, multicentre randomised controlled trial. BMJ.

[41] Hulzebos E.H., Helders P.J., Favie N.J., De Bie R.A., Brutel de la Riviere A., Van Meeteren N.L. (2006). Preoperative intensive inspiratory muscle training to prevent postoperative pulmonary complications in high-risk patients undergoing CABG surgery: a randomized clinical trial. JAMA.

[42] Lawrence V.A., Cornell J.E., Smetana G.W. (2006). American College of P. Strategies to reduce postoperative pulmonary complications after noncardiothoracic surgery: systematic review for the American College of Physicians. Ann Intern Med.

[43] Qaseem A., Snow V., Fitterman N. (2006). Risk assessment for and strategies to reduce perioperative pulmonary complications for patients undergoing noncardiothoracic surgery: a guideline from the American College of Physicians. Ann Intern Med.

[44] Liu Y., Fu M., Zhou Q., Tian M., Zhang X., Wang Z. (2022). The application of patient-centered care bundle significantly reduces incidence of perioperative respiratory complications in hip fracture patients aged 80 and over. Geriatr Nurs.

[45] Choi E.M., Na S., Choi S.H., An J., Rha K.H., Oh Y.J. (2011). Comparison of volume-controlled and pressure-controlled ventilation in steep Trendelenburg position for robot-assisted laparoscopic radical prostatectomy. J Clin Anesth.

[46] Jiang J., Li B., Kang N., Wu A., Yue Y. (2016). Pressure-Controlled Versus Volume-Controlled Ventilation for Surgical Patients: A Systematic Review and Meta-analysis. J Cardiothorac Vasc Anesth.

[47] Liu Z., Liu X., Huang Y., Zhao J. (2016). Intraoperative mechanical ventilation strategies in patients undergoing one-lung ventilation: a meta-analysis. Springerplus.

[48] Cadi P., Guenoun T., Journois D., Chevallier J.M., Diehl J.L., Safran D. (2008). Pressure-controlled ventilation improves oxygenation during laparoscopic obesity surgery compared with volume-controlled ventilation. Br J Anaesth.

[49] Dion J.M., McKee C., Tobias J.D. (2014). Ventilation during laparoscopic-assisted bariatric surgery: volume-controlled, pressure-controlled or volume-guaranteed pressure-regulated modes. Int J Clin Exp Med.

[50] Unzueta M.C., Casas J.I. (2007). Moral MV. Pressure-controlled versus volume-controlled ventilation during one-lung ventilation for thoracic surgery. Anesth Analg.

[51] Snoek K.G., Capolupo I., van Rosmalen J. (2016). Conventional Mechanical Ventilation Versus High-frequency Oscillatory Ventilation for Congenital Diaphragmatic Hernia: A Randomized Clinical Trial (The VICI-trial). Ann Surg.

[52] Yang H.B., Pierro A., Kim HY. (2023). Comparison of conventional mechanical ventilation and high-frequency oscillatory ventilation in congenital diaphragmatic hernias: a systematic review and meta-analysis. Sci Rep.

[53] Batyrkhanov M., Mukhtarkhanova D. (2025). Individualized parameters for mechanical ventilation during thoracic operations: Optimizing respiratory support. Can J Respir Ther.

[54] Young C.C., Harris E.M., Vacchiano C. (2019). Lung-protective ventilation for the surgical patient: international expert panel-based consensus recommendations. Br J Anaesth.

[55] Fogagnolo A., Montanaro F., Al-Husinat L. (2021). Management of Intraoperative Mechanical Ventilation to Prevent Postoperative Complications after General Anesthesia: A Narrative Review. J Clin Med.

[56] Hennessey E., Bittner E., White P., Kovar A., Meuchel L. (2023). Intraoperative Ventilator Management of the Critically Ill Patient. Anesthesiol Clin.

[57] Brochard L., Martin G.S., Blanch L. (2012). Clinical review: Respiratory monitoring in the ICU - a consensus of 16. Crit Care.

[58] Hamahata N.T., Sato R., Daoud EG. (2020). Go with the flow-clinical importance of flow curves during mechanical ventilation: A narrative review. Can J Respir Ther.

[59] Nikolopoulou M.Z., Katsimperis S., Nikolaki D., Kokolaki M. (2024). Flow-Volume Loop: A Tool for Evaluating the Position of the Laryngeal Mask Airway Intraoperatively. Cureus.

[60] Ward L.A., Pezzano C.J., Nathan R.S. (2023). Harwayne-Gidansky I. Use of Flow-Volume Loops on a Mechanically Ventilated Pediatric Patient as a Diagnostic Tool for Fixed Airway Obstruction. Cureus.

[61] Placenti A., Fratebianchi F. (2022). Interpretation and use of intraoperative protective ventilation parameters: a scoping review. Anaesthesiol Intensive Ther.

[62] Tusman G., Bohm S.H., Warner D.O. (2012). Sprung J. Atelectasis and perioperative pulmonary complications in high-risk patients. Curr Opin Anaesthesiol.

[63] Hess DR. (2014). Respiratory mechanics in mechanically ventilated patients. Respir Care.

[64] Gu W.J., Cen Y., Zhao F.Z., Wang H.J., Yin H.Y., Zheng XF. (2024). Association between driving pressure-guided ventilation and postoperative pulmonary complications in surgical patients: a meta-analysis with trial sequential analysis. Br J Anaesth.

[65] Kim Y.J., Kim B.R., Kim H.W. (2023). Effect of driving pressure-guided positive end-expiratory pressure on postoperative pulmonary complications in patients undergoing laparoscopic or robotic surgery: a randomised controlled trial. Br J Anaesth.

[66] Williams E.C., Motta-Ribeiro G.C., Vidal Melo MF. (2019). Driving Pressure and Transpulmonary Pressure: How Do We Guide Safe Mechanical Ventilation?. Anesthesiology.

[67] Karalapillai D., Weinberg L., Neto A.S. (2022). Intra-operative ventilator mechanical power as a predictor of postoperative pulmonary complications in surgical patients: A secondary analysis of a randomised clinical trial. Eur J Anaesthesiol.

[68] Schuijt M.T.U., Hol L., Nijbroek S.G. (2022). Associations of dynamic driving pressure and mechanical power with postoperative pulmonary complications-posthoc analysis of two randomised clinical trials in open abdominal surgery. EClinicalMedicine.

[69] Chen L., Yu K., Yang J. (2024). Electrical impedance tomography-guided positive end-expiratory pressure titration for perioperative oxygenation and postoperative pulmonary complications: A systematic review and meta-analysis. Medicine (Baltimore).

[70] Wu L., Yang L., Yang Y., Wu X., Zhang J. (2024). Ultrasound-guided versus conventional lung recruitment manoeuvres in thoracic surgery: a randomised controlled study. J Clin Monit Comput.

[71] Park S.K., Yang H., Yoo S. (2021). Ultrasound-guided versus conventional lung recruitment manoeuvres in laparoscopic gynaecological surgery: A randomised controlled trial. Eur J Anaesthesiol.

[72] Wu X.Z., Xia H.M., Zhang P. (2022). Effects of ultrasound-guided alveolar recruitment manoeuvres compared with sustained inflation or no recruitment manoeuvres on atelectasis in laparoscopic gynaecological surgery as assessed by ultrasonography: a randomized clinical trial. BMC Anesthesiol.

[73] Gao Y., He H., Chi Y., Frerichs I., Long Y., Zhao Z. (2024). Electrical impedance tomography guided positive end-expiratory pressure titration in critically ill and surgical adult patients: a systematic review and meta-analysis. BMC Pulm Med.

[74] Jiang L., Deng Y., Xu F., Qiao S., Wang C. (2024). Individualized PEEP guided by EIT in patients undergoing general anesthesia: A systematic review and meta-analysis. J Clin Anesth.

[75] Vetrugno L., Deana C., Colaianni-Alfonso N., Tritapepe F., Fierro C., Maggiore SM. (2024). Noninvasive respiratory support in the perioperative setting: a narrative review. Front Med (Lausanne).

[76] Einav S., Lakbar I., Leone M. (2021). Non-Invasive Respiratory Support for Management of the Perioperative Patient: A Narrative Review. Adv Ther.

[77] Elfeky A., Chen Y.F., Grove A. (2025). Perioperative oxygen therapy: an overview of systematic reviews and meta-analyses. Br J Anaesth.

[78] Goret M., Pluchon K., Le Mao R. (2025). Impact of Noninvasive Ventilation Before and After Cardiac Surgery for Preventing Cardiac and Pulmonary Complications: A Clinical Randomized Trial. Chest.

[79] Futier E., Paugam-Burtz C., Godet T. (2016). Effect of early postextubation high-flow nasal cannula vs conventional oxygen therapy on hypoxaemia in patients after major abdominal surgery: a French multicentre randomised controlled trial (OPERA). Intensive Care Med.

[80] Wang Y., Huang D., Ni Y., Liang Z. (2020). High-Flow Nasal Cannula vs Conventional Oxygen Therapy for Postcardiothoracic Surgery. Respir Care.

[81] La Via L., Cuttone G., Senussi Testa T. (2025). Non-Invasive Positive Pressure Ventilation for Pre-Oxygenation of Critically Ill Patients Before Intubation. J Clin Med.

[82] Davidson A.C., Banham S., Elliott M. (2016). BTS/ICS guideline for the ventilatory management of acute hypercapnic respiratory failure in adults. Thorax.

[83] Sequera-Ramos L., Garcia-Marcinkiewicz A., Riva T., Fuchs A. (2022). Noninvasive ventilation in children: A review for the pediatric anesthesiologist. Paediatr Anaesth.

[84] Abrard S., Rineau E., Seegers V. (2023). Postoperative prophylactic intermittent noninvasive ventilation versus usual postoperative care for patients at high risk of pulmonary complications: a multicentre randomised trial. Br J Anaesth.

[85] Hui S., Fowler A.J., Cashmore R.M.J. (2022). Routine postoperative noninvasive respiratory support and pneumonia after elective surgery: a systematic review and meta-analysis of randomised trials. Br J Anaesth.

[86] Hao J., Liu J., Pu L. (2023). High-Flow Nasal Cannula Oxygen Therapy versus Non-Invasive Ventilation in AIDS Patients with Acute Respiratory Failure: A Randomized Controlled Trial. J Clin Med.

[87] Tan D., Walline J.H., Ling B., Xu Y., Sun J., Wang B. (2020). High-flow nasal cannula oxygen therapy versus non-invasive ventilation for chronic obstructive pulmonary disease patients after extubation: a multicenter, randomized controlled trial. Crit Care.

[88] Serpa Neto A., Schultz M.J. (2015). Gama de Abreu M. Intraoperative ventilation strategies to prevent postoperative pulmonary complications: Systematic review, meta-analysis, and trial sequential analysis. Best Pract Res Clin Anaesthesiol.

[89] Tsumura H., Harris E., Brandon D., Pan W., Vacchiano C. (2021). Review of the Mechanisms of Ventilator Induced Lung Injury and the Principles of Intraoperative Lung Protective Ventilation. AANA J.

[90] Nguyen T.K.M., Dinh H., Le A.N. (2021). A review of intraoperative lung-protective mechanical ventilation strategy. Trends Anaesth Crit Care.

[91] Hemmes S.N., Serpa Neto A., Schultz M.J. (2013). Intraoperative ventilatory strategies to prevent postoperative pulmonary complications: a meta-analysis. Curr Opin Anaesthesiol.

[92] Zhang L., Xiong W., Peng Y., Zhang W., Han R. (2018). The effect of an intraoperative, lung-protective ventilation strategy in neurosurgical patients undergoing craniotomy: study protocol for a randomized controlled trial. Trials.

[93] Douville N.J., Jewell E.S., Duggal N. (2020). Association of Intraoperative Ventilator Management With Postoperative Oxygenation, Pulmonary Complications, and Mortality. Anesth Analg.

[94] Ball L., Costantino F., Orefice G., Chandrapatham K., Pelosi P. (2017). Intraoperative mechanical ventilation: state of the art. Minerva Anestesiol.

[95] Ball L., Talmor D., Pelosi P. (2024). Transpulmonary pressure monitoring in critically ill patients: pros and cons. Crit Care.

[96] Guldner A., Kiss T., Serpa Neto A. (2015). Intraoperative protective mechanical ventilation for prevention of postoperative pulmonary complications: a comprehensive review of the role of tidal volume, positive end-expiratory pressure, and lung recruitment maneuvers. Anesthesiology.

[97] Amato M.B., Meade M.O., Slutsky A.S. (2015). Driving pressure and survival in the acute respiratory distress syndrome. N Engl J Med.

[98] Mazzinari G., Zampieri F.G., Ball L. (2025). High Positive End-expiratory Pressure (PEEP) with Recruitment Maneuvers versus Low PEEP during General Anesthesia for Surgery: A Bayesian Individual Patient Data Meta-analysis of Three Randomized Clinical Trials. Anesthesiology.

[99] Mo J., Wang D., Xiao J., Chen Q., An R., Liu H.L. (2024). Effects of lung protection ventilation strategies on postoperative pulmonary complications after noncardiac surgery: a network meta-analysis of randomized controlled trials. BMC Anesthesiol.

[100] Ferrando C., Carraminana A., Pineiro P. (2024). Individualised, perioperative open-lung ventilation strategy during one-lung ventilation (iPROVE-OLV): a multicentre, randomised, controlled clinical trial. Lancet Respir Med.

[101] Anaesthesiology PNIftCTNotESo, Hemmes S.N., Gama de Abreu M., Pelosi P., Schultz M.J. (2014). High versus low positive end-expiratory pressure during general anaesthesia for open abdominal surgery (PROVHILO trial): a multicentre randomised controlled trial. Lancet.

[102] PCGotPVNftCTNotESoA Writing Committee for the, T Bluth, Serpa Neto A., Schultz M.J. (2019). Effect of Intraoperative High Positive End-Expiratory Pressure (PEEP) With Recruitment Maneuvers vs Low PEEP on Postoperative Pulmonary Complications in Obese Patients: A Randomized Clinical Trial. JAMA.

[103] Sun Z., Sessler D.I., Dalton J.E. (2015). Postoperative Hypoxemia Is Common and Persistent: A Prospective Blinded Observational Study. Anesth Analg.

[104] Asfar P., Schortgen F., Boisrame-Helms J. (2017). Hyperoxia and hypertonic saline in patients with septic shock (HYPERS2S): a two-by-two factorial, multicentre, randomised, clinical trial. Lancet Respir Med.

[105] Chu D.K., Kim L.H., Young P.J. (2018). Mortality and morbidity in acutely ill adults treated with liberal versus conservative oxygen therapy (IOTA): a systematic review and meta-analysis. Lancet.

[106] Cumpstey A.F., Oldman A.H., Martin D.S., Smith A., Grocott MPW. (2022). Oxygen Targets During Mechanical Ventilation in the ICU: A Systematic Review and Meta-Analysis. Crit Care Explor.

[107] Douin D.J., Rice J.D., Anderson E.L. (2025). Targeted Normoxemia and Supplemental Oxygen-Free Days in Critically Injured Adults: A Stepped-Wedge Cluster Randomized Clinical Trial. JAMA Netw Open.

[108] Panwar R., Hardie M., Bellomo R. (2016). Conservative versus Liberal Oxygenation Targets for Mechanically Ventilated Patients. A Pilot Multicenter Randomized Controlled Trial. Am J Respir Crit Care Med.

[109] Barrot L., Asfar P., Mauny F. (2020). Liberal or Conservative Oxygen Therapy for Acute Respiratory Distress Syndrome. N Engl J Med.

[110] Suzuki S. (2020). Oxygen administration for postoperative surgical patients: a narrative review. J Intensive Care.

[111] Girardis M., Busani S., Damiani E. (2016). Effect of Conservative vs Conventional Oxygen Therapy on Mortality Among Patients in an Intensive Care Unit: The Oxygen-ICU Randomized Clinical Trial. JAMA.

[112] Mackle D., Bellomo R., Bailey M., Investigators I-R, the A, New Zealand Intensive Care Society Clinical Trials G (2020). Conservative Oxygen Therapy during Mechanical Ventilation in the ICU. N Engl J Med..

[113] Schjorring O.L., Klitgaard T.L., Perner A. (2021). Lower or Higher Oxygenation Targets for Acute Hypoxemic Respiratory Failure. N Engl J Med.

[114] Jaju R., Jaju P.B., Dubey M., Mohammad S., Bhargava AK. (2017). Comparison of volume controlled ventilation and pressure controlled ventilation in patients undergoing robot-assisted pelvic surgeries: An open-label trial. Indian J Anaesth.

[115] Wang J.P., Wang H.B., Liu Y.J., Lou X.P., Wang X.D., Kong Y. (2015). Comparison of Pressure- and Volume-Controlled Ventilation in Laparoscopic Surgery: A Meta-analysis of Randomized Controlled Trial. Clin Invest Med.

[116] Chan K., Abraham E. (1992). Effects of inverse ratio ventilation on cardiorespiratory parameters in severe respiratory failure. Chest.

[117] Poelaert J.I., Visser C.A., Everaert J.A., Koolen J.J., Colardyn FA. (1993). Acute hemodynamic changes of pressure-controlled inverse ratio ventilation in the adult respiratory distress syndrome. A transesophageal echocardiographic and Doppler study. Chest.

[118] Lessard M.R., Guerot E., Lorino H., Lemaire F., Brochard L. (1994). Effects of pressure-controlled with different I:E ratios versus volume-controlled ventilation on respiratory mechanics, gas exchange, and hemodynamics in patients with adult respiratory distress syndrome. Anesthesiology.

[119] Takahashi Y., Moriyama K., Kubota H. (2023). [Obstructive Lung Disease:Point of Perioperative Management]. Kyobu Geka.

[120] Jia Y., Leung S.M., Turan A. (2020). Low Tidal Volumes Are Associated With Slightly Improved Oxygenation in Patients Having Cardiac Surgery: A Cohort Analysis. Anesth Analg.

[121] Peigang Y., Marini JJ. (2002). Ventilation of patients with asthma and chronic obstructive pulmonary disease. Curr Opin Crit Care.

[122] Leatherman J.W., McArthur C., Shapiro RS. (2004). Effect of prolongation of expiratory time on dynamic hyperinflation in mechanically ventilated patients with severe asthma. Crit Care Med.

[123] Laghi F., Goyal A. (2012). Auto-PEEP in respiratory failure. Minerva Anestesiol.

[124] Reddy VG. (2005). Auto-PEEP: how to detect and how to prevent–a review. Middle East J Anaesthesiol.

[125] Tuxen D.V., Williams T.J., Scheinkestel C.D., Czarny D., Bowes G. (1992). Use of a measurement of pulmonary hyperinflation to control the level of mechanical ventilation in patients with acute severe asthma. Am Rev Respir Dis.

[126] Guerin C., Lemasson S., La Cara M.F., Fournier G. (2002). Physiological effects of constant versus decelerating inflation flow in patients with chronic obstructive pulmonary disease under controlled mechanical ventilation. Intensive Care Med.

[127] Kallet R.H., Campbell A.R., Alonso J.A., Morabito D.J., Mackersie RC. (2000). The effects of pressure control versus volume control assisted ventilation on patient work of breathing in acute lung injury and acute respiratory distress syndrome. Respir Care.

[128] Douville N.J., McMurry T.L., Ma J.Z. (2022). Airway driving pressure is associated with postoperative pulmonary complications after major abdominal surgery: a multicentre retrospective observational cohort study. BJA Open.

[129] Pirrone M., Fisher D., Chipman D. (2016). Recruitment Maneuvers and Positive End-Expiratory Pressure Titration in Morbidly Obese ICU Patients. Crit Care Med.

[130] Sumer I., Topuz U., Alver S. (2020). Effect of the "Recruitment" Maneuver on Respiratory Mechanics in Laparoscopic Sleeve Gastrectomy Surgery. Obes Surg.

[131] Hu M.C., Yang Y.L., Chen T.T., Lee C.I., Tam KW. (2022). Recruitment maneuvers to reduce pulmonary atelectasis after cardiac surgery: A meta-analysis of randomized trials. J Thorac Cardiovasc Surg.

[132] Pritchett M.A., Lau K., Skibo S., Phillips K.A., Bhadra K. (2021). Anesthesia considerations to reduce motion and atelectasis during advanced guided bronchoscopy. BMC Pulm Med.

[133] Wang H., Wang Z., Wu Q. (2025). Perioperative oxygen administration for adults undergoing major noncardiac surgery: a narrative review. Med Gas Res.

[134] Wamsley C., Missel D. (2016). Effect of peak inspiratory pressure on the development of postoperative pulmonary complications in mechanically ventilated adult surgical patients: a systematic review protocol. JBI Database System Rev Implement Rep.

[135] Silva P.L., Rocco PRM. (2018). The basics of respiratory mechanics: ventilator-derived parameters. Ann Transl Med.

[136] Umbrello M., Chiumello D. (2018). Interpretation of the transpulmonary pressure in the critically ill patient. Ann Transl Med.

[137] Boesing C., Schaefer L., Hammel M. (2023). Individualized Positive End-expiratory Pressure Titration Strategies in Superobese Patients Undergoing Laparoscopic Surgery: Prospective and Nonrandomized Crossover Study. Anesthesiology.

[138] Fernandez-Bustamante A., Hashimoto S., Serpa Neto A., Moine P., Vidal Melo M.F., Repine J.E. (2015). Perioperative lung protective ventilation in obese patients. BMC Anesthesiol.

[139] Jimenez-Santana JD D-CO, Schultz M.J., Mazzinari G. (2024). Current Concepts in Intraoperative Ventilation during Anesthesia for Laparoscopic and Robot–Assisted Surgery – a Narrative Review. Curr Anesthesiol Rep.

[140] Pozzi T., Coppola S., Catozzi G. (2024). Mechanical power during robotic-assisted laparoscopic prostatectomy: an observational study. J Clin Monit Comput.

[141] Nguyen T.K., Nguyen V.L., Nguyen T.G. (2021). Lung-protective mechanical ventilation for patients undergoing abdominal laparoscopic surgeries: a randomized controlled trial. BMC Anesthesiol.

[142] Sun M., Jia R., Wang L. (2023). Effect of protective lung ventilation on pulmonary complications after laparoscopic surgery: a meta-analysis of randomized controlled trials. Front Med (Lausanne).

[143] Choi S., Yang S.Y., Choi G.J., Kim B.G., Kang H. (2019). Comparison of pressure- and volume-controlled ventilation during laparoscopic colectomy in patients with colorectal cancer. Sci Rep.

[144] Schick V., Dusse F., Eckardt R. (2021). Comparison of Volume-Guaranteed or -Targeted, Pressure-Controlled Ventilation with Volume-Controlled Ventilation during Elective Surgery: A Systematic Review and Meta-Analysis. J Clin Med.

[145] Turan Civraz A.Z., Saracoglu A., Saracoglu K.T. (2023). Evaluation of the Effect of Pressure-Controlled Ventilation-Volume Guaranteed Mode vs. Volume-Controlled Ventilation Mode on Atelectasis in Patients Undergoing Laparoscopic Surgery: A Randomized Controlled Clinical Trial. Medicina (Kaunas)..

[146] Sevdi M.S., Demirgan S., Erkalp K. (2021). Comparison of Intra-operative Pressure-Controlled Ventilation and Volume-Controlled Ventilation in Bariatric Surgery: A Prospective Randomized Study. Cureus.

[147] Buonanno P., Marra A., Iacovazzo C. (2023). Electric impedance tomography and protective mechanical ventilation in elective robotic-assisted laparoscopy surgery with steep Trendelenburg position: a randomized controlled study. Sci Rep.

[148] Yang G., Zhang P., Li L. (2023). Driving Pressure-Guided Ventilation in Obese Patients Undergoing Laparoscopic Sleeve Gastrectomy: A Randomized Controlled Trial. Diabetes Metab Syndr Obes.

[149] Bae Y.K., Nam S.W., Oh A.Y. (2024). Effect of the alveolar recruitment maneuver during laparoscopic colorectal surgery on postoperative pulmonary complications: A randomized controlled trial. PLoS One.

[150] Dargvainis M., Ohnesorge H., Schadler D., Alkatout I., Frerichs I., Becher T. (2022). Recruitable alveolar collapse and overdistension during laparoscopic gynecological surgery and mechanical ventilation: a prospective clinical study. BMC Anesthesiol.

[151] Asehnoune K., Rooze P., Robba C. (2023). Mechanical ventilation in patients with acute brain injury: a systematic review with meta-analysis. Crit Care.

[152] Jiang L., Wu Y., Zhang Y., Lu D., Yan K., Gao J. (2021). Effects of intraoperative lung-protective ventilation on clinical outcomes in patients with traumatic brain injury: a randomized controlled trial. BMC Anesthesiol.

[153] Mascia L., Fanelli V., Mistretta A. (2024). Lung-Protective Mechanical Ventilation in Patients with Severe Acute Brain Injury: A Multicenter Randomized Clinical Trial (PROLABI). Am J Respir Crit Care Med.

[154] Bossers S.M., Mansvelder F., Loer S.A. (2023). Association between prehospital end-tidal carbon dioxide levels and mortality in patients with suspected severe traumatic brain injury. Intensive Care Med.

[155] Robba C., Battaglini D., Abbas A. (2024). Clinical practice and effect of carbon dioxide on outcomes in mechanically ventilated acute brain-injured patients: a secondary analysis of the ENIO study. Intensive Care Med.

[156] Giardina A., Cardim D., Ciliberti P. (2023). Effects of positive end-expiratory pressure on cerebral hemodynamics in acute brain injury patients. Front Physiol.

[157] Grande B., Ganter MT. (2018). What is the best strategy for one-lung ventilation during thoracic surgery?. J Thorac Dis.

[158] El Tahan M.R., Samara E., Marczin N., Landoni G., Pasin L. (2023). Impact of Lower Tidal Volumes During One-Lung Ventilation: A 2022 Update of the Meta-analysis of Randomized Controlled Trials. J Cardiothorac Vasc Anesth.

[159] Peel J.K., Funk D.J., Slinger P., Srinathan S., Kidane B. (2022). Tidal volume during 1-lung ventilation: A systematic review and meta-analysis. J Thorac Cardiovasc Surg.

[160] Kiss T., Wittenstein J., Becker C. (2019). Protective ventilation with high versus low positive end-expiratory pressure during one-lung ventilation for thoracic surgery (PROTHOR): study protocol for a randomized controlled trial. Trials.

[161] Li P., Kang X., Miao M., Zhang J. (2021). Individualized positive end-expiratory pressure (PEEP) during one-lung ventilation for prevention of postoperative pulmonary complications in patients undergoing thoracic surgery: A meta-analysis. Medicine (Baltimore).

[162] Kim H. (2014). Protective strategies for one-lung ventilation. Korean J Anesthesiol.

[163] Meleiro H., Correia I., Charco Mora P. (2018). New evidence in one-lung ventilation. Rev Esp Anestesiol Reanim (Engl Ed).

[164] Kim K.N., Kim D.W., Jeong M.A., Sin Y.H., Lee SK. (2016). Comparison of pressure-controlled ventilation with volume-controlled ventilation during one-lung ventilation: a systematic review and meta-analysis. BMC Anesthesiol.

[165] Gao W., Liu D.D., Li D., Cui G.X. (2015). Effect of Therapeutic Hypercapnia on Inflammatory Responses to One-lung Ventilation in Lobectomy Patients. Anesthesiology.

[166] Roze H., Lafargue M., Perez P. (2012). Reducing tidal volume and increasing positive end-expiratory pressure with constant plateau pressure during one-lung ventilation: effect on oxygenation. Br J Anaesth.

[167] Spadaro S., Grasso S., Karbing D.S. (2021). Physiological effects of two driving pressure-based methods to set positive end-expiratory pressure during one lung ventilation. J Clin Monit Comput.

[168] Koo C.H., Park E.Y., Lee S.Y., Ryu JH. (2019). The Effects of Intraoperative Inspired Oxygen Fraction on Postoperative Pulmonary Parameters in Patients with General Anesthesia: A Systemic Review and Meta-Analysis. J Clin Med.

[169] Rawley M., Harris E., Pospishil L., Thompson J.A., Falyar C. (2022). Assessing Provider Adherence To A Lung Protective Ventilation Protocol In Patients Undergoing Thoracic Surgery Using One-Lung Ventilation. AANA J.

[170] Kozian A., Schilling T. (2014). Protective Ventilatory Approaches to One-Lung Ventilation: More than Reduction of Tidal Volume. Current Anesthesiology Reports.

[171] Lagier D., Fischer F., Fornier W. (2019). Effect of open-lung vs conventional perioperative ventilation strategies on postoperative pulmonary complications after on-pump cardiac surgery: the PROVECS randomized clinical trial. Intensive Care Med.

[172] Lagier D., Velly L.J., Guinard B. (2020). Perioperative Open-lung Approach, Regional Ventilation, and Lung Injury in Cardiac Surgery. Anesthesiology.

[173] Longo S., Siri J., Acosta C. (2017). Lung recruitment improves right ventricular performance after cardiopulmonary bypass: A randomised controlled trial. Eur J Anaesthesiol.

[174] Serita R., Morisaki H., Takeda J. (2009). An individualized recruitment maneuver for mechanically ventilated patients after cardiac surgery. J Anesth.

[175] Petrucci N., Iacovelli W. (2004). Ventilation with lower tidal volumes versus traditional tidal volumes in adults for acute lung injury and acute respiratory distress syndrome. Cochrane Database Syst Rev.

[176] Rodrigues R.R., Sawada A.Y., Rouby J.J. (2011). Computed tomography assessment of lung structure in patients undergoing cardiac surgery with cardiopulmonary bypass. Braz J Med Biol Res.

[177] Heath C., Hauser N. (2022). Is there a role for lung-protective ventilation in healthy children?. Paediatr Anaesth.

[178] Bruins S., Sommerfield D., Powers N. (2022). von Ungern-Sternberg BS. Atelectasis and lung recruitment in pediatric anesthesia: An educational review. Paediatr Anaesth.

[179] Kneyber M.C.J., de Luca D., Calderini E. (2017). Recommendations for mechanical ventilation of critically ill children from the Paediatric Mechanical Ventilation Consensus Conference (PEMVECC). Intensive Care Med.

[180] Feldman JM. (2015). Optimal ventilation of the anesthetized pediatric patient. Anesth Analg.

[181] Dos Santos Rocha A., Habre W., Albu G. (2022). Novel ventilation techniques in children. Paediatr Anaesth.

[182] Jeong H., Tanatporn P., Ahn H.J. (2021). Pressure Support versus Spontaneous Ventilation during Anesthetic Emergence-Effect on Postoperative Atelectasis: A Randomized Controlled Trial. Anesthesiology.

[183] Rimensberger P.C., Cheifetz I.M. (2015). Pediatric Acute Lung Injury Consensus Conference G. Ventilatory support in children with pediatric acute respiratory distress syndrome: proceedings from the Pediatric Acute Lung Injury Consensus Conference. Pediatr Crit Care Med..

[184] Spaeth J., Schumann S., Humphreys S. (2022). Understanding pediatric ventilation in the operative setting. Part II: Setting perioperative ventilation. Paediatr Anaesth.

[185] Acosta C.M., Sara T., Carpinella M. (2018). Lung recruitment prevents collapse during laparoscopy in children: A randomised controlled trial. Eur J Anaesthesiol.

[186] Jang Y.E., Ji S.H., Kim E.H., Lee J.H., Kim J.T., Kim HS. (2020). Effect of regular alveolar recruitment on intraoperative atelectasis in paediatric patients ventilated in the prone position: a randomised controlled trial. Br J Anaesth.

[187] Acosta C.M., Maidana G.A., Jacovitti D. (2014). Accuracy of transthoracic lung ultrasound for diagnosing anesthesia-induced atelectasis in children. Anesthesiology.

[188] Grandville B., Petak F., Albu G., Bayat S., Pichon I., Habre W. (2019). High inspired oxygen fraction impairs lung volume and ventilation heterogeneity in healthy children: a double-blind randomised controlled trial. Br J Anaesth.

[189] von Ungern-Sternberg BS. (2008). Impact of anaesthesia on lung function in children. European Respiratory Review.

