# Supplementary material for: Consensus recommendations on perioperative mechanical ventilation strategies: an evidence-based guideline by the Brazilian Society of Anesthesiology
Source: Braz J Anesthesiol. 2026 Aug 18;76(5):844814. doi: 10.1016/j.bjane.2026.844814 (PMC13573195; doi:10.1016/j.bjane.2026.844814)
Supplement: Supplementary file 1 [file mmc1.docx]

**BJAN-D-25-00672_Supplementary Material**

**Supplementary Table S1** Results of expert voting on guideline topics (Delphi method).

| **Topic** | **Question** | **GRADE** | **Evidence Quality** | **Agreement (%)** | **Disagreement (%)** |
| --- | --- | --- | --- | --- | --- |
| 1 - Definition of Postoperative Pulmonary Complications in Surgical Patients | PPCs should be defined using standardized clinical, radiological, and laboratory criteria. | Strong | High | 100.0 | 0.0 |
|  | Definitions should rely on objective parameters to allow reproducibility and comparability across studies. | Strong | Moderate | 100.0 | 0.0 |
|  | A core set of complications, such as respiratory failure, pleural effusion, pneumothorax, atelectasis, bronchospasm, aspiration pneumonitis, pneumonia, and ARDS should be routinely assessed. | Strong | High | 96.7 | 3.3 |
|  | Using harmonized definitions improves benchmarking, outcome tracking, and implementation of protective strategies. | Strong | Moderate | 100.0 | 0.0 |
| 2 - Postoperative Pulmonary Complications: Risk Factors and Preventive Strategies | Preoperative risk stratification using validated tools (e.g., ARISCAT score) is recommended to identify patients at high risk for PPCs. | Strong | High | 100.0 | 0.0 |
|  | Optimization of comorbidities (e.g., COPD, heart failure, malnutrition, anemia) should be undertaken before surgery whenever possible. | Strong | Moderate | 100.0 | 0.0 |
|  | Smoking cessation at least 4–8 weeks before surgery is recommended to reduce the risk of PPCs. | Strong | Moderate | 100.0 | 0.0 |
|  | Supervised respiratory physiotherapy and early mobilization (including preoperative prehabilitation for thoracic/cardiac surgery and supervised postoperative therapy in high-risk patients). | Weak | Moderate | 96.7 | 3.3 |
|  | Minimally invasive surgical approaches (e.g., laparoscopy) are associated with reduced PPCs when feasible. | Strong | Moderate | 96.7 | 3.3 |
|  | Bundle-based preventive strategies (multimodal perioperative protocols) are encouraged to maximize impact on reducing PPCs. | Strong | Moderate | 100.0 | 0.0 |
| 3 - Ventilation Modes and Intraoperative Considerations | Volume-Controlled Ventilation (VCV) and Pressure-Controlled Ventilation (PCV) provide comparable outcomes when protective settings are applied. | Strong | Moderate | 100.0 | 0.0 |
|  | Dual-control modes (e.g., PCV-VG) may be considered to optimize tidal volume delivery in patients with variable compliance, particularly in obesity and laparoscopy. | Weak | Low | 96.7 | 3.3 |
|  | Use pressure-controlled ventilation when peak airway pressures are a concern, recognizing that this strategy may limit peak pressure but at the expense of tidal volume delivery. | Weak | Low | 86.7 | 13.3 |
|  | Avoid high-frequency oscillatory ventilation or unconventional modes intraoperatively outside research settings. | Strong | Moderate | 100.0 | 0.0 |
|  | In patients with compromised respiratory mechanics, individualized mode selection is encouraged. | Strong | Low | 96.7 | 3.3 |
| 4 - Monitoring of Intraoperative Mechanical Ventilation | Suggest monitoring the pressure-time curve to detect overdistension or alveolar recruitment using the stress index (SI = 1). | Weak | Low | 96.7 | 3.3 |
|  | Recommend using the flow-time curve to detect auto-PEEP, especially in high respiratory rate situations. | Strong | Very Low | 96.7 | 3.3 |
|  | Pressure-volume and flow-volume loops may aid in assessing compliance and resistance. | Weak | Very Low | 96.7 | 3.3 |
|  | Recommend monitoring plateau pressure and maintaining it below 25–30 cm H₂O. | Strong | Very Low | 93.3 | 6.7 |
|  | Suggest interpreting plateau pressure with caution in cases of altered chest wall mechanics. | Weak | Very Low | 96.7 | 3.3 |
|  | Transpulmonary pressure monitoring may be useful in individualizing PEEP, especially in complex cases. | Weak | Very Low | 93.3 | 6.7 |
|  | Driving pressure (ΔP) should be monitored, aiming for values ≤ 15 cm H_2_O, ideally ≤ 12 cm H_2_O. | Strong | Moderate | 96.7 | 3.3 |
|  | Mechanical power monitoring may help adjust ventilatory parameters to reduce PPCs. | Weak | Low | 96.7 | 3.3 |
| 5 - Advanced Monitoring of Intraoperative Ventilation | Use of LUS to guide alveolar recruitment is suggested in laparoscopic or thoracoscopic surgery. | Strong | Moderate | 90.0 | 10.0 |
|  | EIT may be used intraoperatively to titrate PEEP and improve oxygenation and compliance. | Weak | Moderate | 100.0 | 0.0 |
| 6 - Perioperative Use of Non-Invasive Ventilation (NIV) and High-Flow Nasal Cannula (HFNC) | NIV is recommended for the treatment of acute postoperative hypoxemic respiratory failure in cooperative, hemodynamically stable patients. | Strong | High | 100.0 | 0.0 |
|  | HFNC is recommended as an alternative to conventional oxygen therapy after extubation in high-risk surgical patients to improve oxygenation and comfort. | Strong | Moderate | 100.0 | 0.0 |
|  | NIV or HFNC may be considered for preoxygenation before intubation in obese or hypoxemic patients to prolong safe apnea time and reduce desaturation. | Weak | Moderate | 100.0 | 0.0 |
|  | NIV is not recommended in patients with contraindications such as airway obstruction, hemodynamic instability, impaired consciousness, or copious secretions. | Strong | High | 96.7 | 3.3 |
|  | Routine prophylactic use of NIV in unselected postoperative patients is not recommended, especially after abdominal or thoracic surgery. | Strong | High | 100.0 | 0.0 |
|  | HFNC may be considered in patients intolerant to NIV interfaces or when NIV is contraindicated. | Weak | Low | 96.7 | 3.3 |
| 7 - Intraoperative Mechanical Ventilation Strategies | Use tidal volumes of 6–8 mL.kg^-1^ PBW for all patients, regardless of actual weight. | Strong | High | 96.7 | 3.3 |
|  | Avoid tidal volumes > 10 mL.kg^-1^ PBW. | Strong | High | 100.0 | 0.0 |
|  | Maintain plateau pressure ≤ 30 cm H_2_O. | Strong | High | 96.7 | 3.3 |
|  | Target driving pressure ≤ 15 cm H_2_O, ideally ≤ 12 cm H_2_O. | Strong | Moderate | 96.7 | 3.3 |
|  | PEEP: Use individualized PEEP titrated to oxygenation and respiratory mechanics (ΔP/compliance); avoid routine ZEEP in higher-risk contexts. | Strong | Moderate | 100.0 | 0.0 |
|  | Favor early extubation in stable patients. | Strong | High | 100.0 | 0.0 |
|  | Consider individualized PEEP guided by physiological signals (e.g., esophageal pressure or EIT) in higher-risk settings (obesity, pneumoperitoneum/Trendelenburg). | Weak | Low | 96.7 | 3.3 |
| 8 - Recruitment Maneuvers | Use recruitment maneuvers in combination with low tidal volume ventilation and individualized PEEP in high-risk patients. | Weak | Moderate | 96.7 | 3.3 |
|  | Apply recruitment maneuvers in cases of intraoperative hypoxemia or suspected reversible atelectasis. | Weak | Moderate | 100.0 | 0.0 |
|  | Avoid routine high-pressure RMs (≥ 40 cm H_2_O for 30s) in all patients, especially without hemodynamic monitoring. | Strong | Moderate | 96.7 | 3.3 |
| 9 - Oxygenation Targets | Maintain SpO_2_ between 92% and 97% during the perioperative period. | Weak | Moderate | 96.7 | 3.3 |
|  | Avoid liberal oxygen therapy (SpO_2_ ≥ 98%) when there is no hypoxemia. | Weak | Moderate | 96.7 | 3.3 |
|  | Avoid targeting SpO_2_ < 90% intra- and postoperatively due to the increased risk of mortality. | Strong | Moderate | 100.0 | 0.0 |
| 10 - Cardiopulmonary Interactions during Intraoperative Ventilation | Pressure-Controlled Ventilation (PCV) and Volume-Controlled Ventilation (VCV) have similar hemodynamic effects when airway pressures are matched. | Strong | Moderate | 100.0 | 0.0 |
|  | Consider PC-IRV instead of VCV to improve oxygenation and reduce peak inspiratory pressure in selected patients. | Weak | Low | 86.7 | 13.3 |
|  | Consider PC-IRV over PCV in ARDS patients to enhance oxygenation and CO_2_ clearance, acknowledging the potential for reduced cardiac output. | Weak | Low | 93.3 | 6.7 |
| **Topic** | **Question** | **GRADE** | **Evidence Quality** | **Agreement (%)** | **Disagreement (%)** |
| 11 - Ventilation in Obstructive Lung Disease | Use low tidal volumes (6–8 mL.kg^-1^ PBW) and prolonged expiratory times to minimize dynamic hyperinflation. | Strong | Moderate | 100.0 | 0.0 |
|  | Maintain low respiratory rates to increase expiratory time and reduce air trapping. | Strong | Moderate | 96.7 | 3.3 |
|  | Monitor for auto-PEEP and consider external PEEP ≤ 80% of measured auto-PEEP only if triggering is compromised. | Conditional | Low | 86.7 | 13.3 |
|  | Avoid high PEEP and recruitment maneuvers unless clear evidence of atelectasis-induced hypoxemia. | Strong | Low | 93.3 | 6.7 |
|  | Use Pressure-Controlled Ventilation (PCV) or Volume-Controlled Ventilation (VCV) with decelerating flow pattern for optimal control of peak pressure. | Conditional | Low | 100.0 | 0.0 |
|  | Adjust inspiratory-to-expiratory (I:E) ratio (e.g., 1:3 to 1:4) and reduce inspiratory flow to minimize air trapping. | Strong | Moderate | 86.7 | 13.3 |
|  | Evaluate plateau pressure and driving pressure to detect early signs of dynamic hyperinflation. | Conditional | Low | 100.0 | 0.0 |
| 12 - Ventilation in Obese Patients | Use tidal volumes between 6–8 mL.kg^-1^ of Predicted Body Weight (PBW), based on the ARDSNet formula, regardless of actual body weight. | Strong | High | 100.0 | 0.0 |
|  | Consider recruitment maneuvers (RM) in cases of persistent hypoxemia (SpO_2_ < 94%). | Weak | Low | 83.3 | 16.7 |
|  | Consider performing RM during PEEP titration to identify the PEEP associated with better compliance or lower driving pressure. | Conditional | Low | 100.0 | 0.0 |
|  | Prefer ventilator-guided RM over manual “bag-squeeze” techniques. | Strong | Moderate | 100.0 | 0.0 |
|  | Use a reduced FiO_2_ (e.g., 0.4–0.6) during RM to minimize reabsorption atelectasis and oxidative stress. | Strong | High | 93.3 | 6.7 |
|  | In morbidly obese patients, plateau pressures up to 40 cm H_2_O may be required during RM, but this carries risk of barotrauma and hemodynamic compromise and requires close monitoring. | Conditional | Moderate | 96.7 | 3.3 |
|  | Prefer Pressure-Controlled Ventilation with Volume Guarantee (PCV-VG) when available, especially in laparoscopic procedures. | Conditional | Moderate | 96.7 | 3.3 |
|  | Consider conventional pressure-controlled ventilation when PCV-VG is unavailable, acknowledging potential tidal volume variability. | Weak | Low | 86.7 | 13.3 |
|  | Volume-controlled ventilation is also acceptable when PCV-VG is unavailable and fixed tidal volume delivery is a priority. | Weak | Low | 93.3 | 6.7 |
|  | Maintain peak airway pressure (Ppeak) < 35 cm H_2_O when possible. | Strong | High | 93.3 | 6.7 |
|  | Maintain plateau pressure (Pplat) ≤ 30 cm H_2_O when possible. | Strong | High | 96.7 | 3.3 |
|  | In specialized centers, consider using estimated transpulmonary pressure (via esophageal balloon) to guide fine-tuning of ventilation in superobese patients (BMI > 50). | Conditional | Low | 96.7 | 3.3 |
|  | Aim to reduce driving pressure (ΔP) and maintain values ≤ 15 cm H_2_O. | Strong | Low | 93.3 | 6.7 |
|  | Maintain PEEP ≥ 5 cm H_2_O to optimize PaO_2_/FiO_2_ ratio intraoperatively. | Strong | High | 96.7 | 3.3 |
|  | Consider titrating PEEP to ≥ 10 cm H_2_O to optimize PaO_2_/FiO_2_ ratio and improve compliance. | Strong | High | 96.7 | 3.3 |
|  | Avoid low PEEP levels (< 5 cm H_2_O) in obese patients; individualized PEEP is safe when paired with adequate hemodynamic monitoring. | Strong | High | 100.0 | 0.0 |
| 13 - Mechanical Ventilation During Laparoscopic Surgery | Use protective ventilation (6–8 mL.kg^-1^ predicted body weight + PEEP) to reduce postoperative complications. | Strong | High | 100.0 | 0.0 |
|  | Avoid conventional ventilation (8–10 mL.kg^-1^, no PEEP) due to increased complications and poorer oxygenation. | Strong | High | 93.3 | 6.7 |
|  | Consider using PCV-VG over VCV to reduce airway pressures and improve compliance. | Weak | Low | 93.3 | 6.7 |
|  | PCV can also be considered in selected patients to reduce inflammatory response, though supporting evidence remains limited. | Weak | Low | 80.0 | 20.0 |
|  | Prefer individualized PEEP titration (guided by ΔP, compliance, or EIT) over fixed PEEP (e.g., 5 cm H_2_O or ZEEP). | Strong | High | 93.3 | 6.7 |
|  | Consider alveolar Recruitment Maneuvers (RMs) to restore compliance, especially after pneumoperitoneum deflation. | Weak | Moderate | 96.7 | 3.3 |
| 14 - Ventilation in Neurosurgical Patients | Use protective ventilation (VT 6–8 mL.kg^-1^ PBW + PEEP ≈5 cm H_2_O) as the standard strategy. | Strong | High | 100.0 | 0.0 |
|  | Avoid high tidal volumes (> 10 mL.kg^-1^ PBW) and ZEEP ventilation. | Strong | High | 96.7 | 3.3 |
|  | Avoid permissive hypercapnia (PaCO_2_ > 45 mmHg) in patients with elevated ICP or altered autoregulation. | Strong | Moderate | 100.0 | 0.0 |
|  | Maintain normocapnia (PaCO_2_ 35–40 mmHg), especially in patients with elevated ICP. | Strong | Moderate | 96.7 | 3.3 |
|  | In patients with invasive neuromonitoring (ICP, PbtO_2_), consider individualized CO_2_ targets guided by cerebral parameters. | Conditional | Low | 100.0 | 0.0 |
| 15 - Ventilation in Thoracic Surgery (One-Lung Ventilation) | Use low tidal volumes (4–6 mL.kg^-1^ PBW) during OLV to reduce lung stress and prevent VILI. | Strong | High | 100.0 | 0.0 |
|  | Apply moderate PEEP (5–10 cm H_2_O) to the dependent lung to prevent atelectasis during OLV. | Strong | Moderate | 96.7 | 3.3 |
|  | Avoid routine use of ZEEP during OLV due to increased risk of alveolar collapse. | Strong | Moderate | 96.7 | 3.3 |
|  | Prefer Pressure-Controlled Ventilation (PCV) or volume-guaranteed pressure modes to limit peak pressure. | Conditional | Moderate | 100.0 | 0.0 |
|  | Maintain plateau pressure ≤ 30 cm H_2_O during OLV. | Strong | High | 90.0 | 10.0 |
|  | Consider individualized PEEP titration based on driving pressure or compliance. | Conditional | Low | 100.0 | 0.0 |
|  | Avoid hyperoxia and unnecessary high FiO_2_; aim for SpO₂ between 92%–96%. | Strong | Moderate | 100.0 | 0.0 |
|  | Recruitment maneuvers may be considered as rescue therapy for severe hypoxemia, to be used cautiously and not routinely. | Weak | Low | 96.6 | 3.4 |
| 16 - Ventilation in Cardiac Surgery | Perform alveolar recruitment maneuvers at the end of CPB to re-expand collapsed lung regions. | Strong | Moderate | 96.7 | 3.3 |
|  | Use individualized PEEP titration (e.g., compliance or driving pressure-based) after RM to maintain alveolar patency, specifying available tools (compliance/ΔP or EIT). | Weak | Moderate | 96.7 | 3.3 |
|  | Avoid high fixed PEEP levels without prior RM due to potential overdistension and hemodynamic compromise. | Strong | Moderate | 100.0 | 0.0 |
|  | Use protective ventilation (6–8 mL.kg^-1^ PBW, Pplat ≤ 30 cm H_2_O) throughout surgery, including before and after CPB. | Strong | Low | 93.3 | 6.7 |
| 17 - Ventilation in Pediatric Patients | Use pressure-controlled ventilation with volume guarantee (PCV-VG) in healthy pediatric patients. | Weak | Moderate | 96.7 | 3.3 |
|  | Consider Pressure Support Ventilation (PSV) with PEEP to maintain spontaneous breathing. | Conditional | Moderate | 100.0 | 0.0 |
|  | Avoid spontaneous ventilation without ventilatory support. | Strong | Moderate | 96.7 | 3.3 |
|  | Use tidal volumes of 5–8 mL.kg^-1^ of ideal body weight. | Conditional | Low | 96.7 | 3.3 |
|  | Avoid tidal volumes > 10 mL.kg^-1^. | Strong | High | 100.0 | 0.0 |
|  | Plateau pressure ≤ 28 cm H_2_O in healthy lungs or ≤ 32 cm H_2_O in obstructive disease. | Conditional | Moderate | 96.7 | 3.3 |
|  | Maintain driving pressure ≤ 15 cm H_2_O. | Conditional | Moderate | 100.0 | 0.0 |
|  | Perform intermittent alveolar recruitment maneuvers, particularly after induction or disconnection. | Strong | High | 93.3 | 6.7 |
|  | Combine recruitment maneuvers with PEEP. | Conditional | High | 93.3 | 6.7 |
|  | Use bedside lung ultrasound to guide recruitment strategies and detect atelectasis. | Conditional | Moderate | 100.0 | 0.0 |
|  | Avoid routine use of FiO_2_ = 1.0 due to risk of absorption atelectasis and heterogeneous ventilation. | Strong | High | 100.0 | 0.0 |
|  | Prefer FiO_2_ < 0.8 throughout the perioperative period, titrated according to need. | Conditional | Moderate | 100.0 | 0.0 |
|  | Consider use of PEEP or CPAP during induction and emergence to reduce atelectasis. | Conditional | Low | 96.7 | 3.3 |

% Agreement, Experts in favor; % Disagreement Experts against; Abstention, No response (if applicable). GRADE, *Grading of Recommendations, Assessment, Development and Evaluation*; QoE, *Quality of Evidence* (high, moderate, low, very low); Strength, Recommendation strength (*Strong* or *Weak/Conditional*). Results derived from Delphi consensus rounds.

**Supplementary Methods Appendix**

**Methodological details for evidence synthesis and grading**

**Search strategy and data sources**

For each PICO question, a structured literature search was performed in MEDLINE (via PubMed), Embase, the Cochrane Library, and Scopus. Search strategies were tailored to the specific population, intervention, and outcomes of interest for each topic. Searches included randomized controlled trials, meta-analyses, observational studies, and high-quality systematic reviews. The final search was updated through July 2025, corresponding to the evidence base considered during guideline development. Reference lists of key articles and guidelines were manually screened to identify additional relevant studies.

**Eligibility criteria**

Studies were considered eligible if they met the following criteria:

• Adult or pediatric surgical patients undergoing perioperative mechanical ventilation.

• Evaluation of ventilatory strategies, monitoring, or respiratory support relevant to the perioperative period.

• Reporting of clinical, physiological, or patient centered outcomes related to pulmonary complications, oxygenation, or ventilator associated harm.

Exclusion criteria included:

• Non-surgical populations without perioperative context.

• Case reports, narrative reviews without systematic methodology, and conference abstracts without full data.

• Studies focusing exclusively on long term intensive care ventilation unrelated to the perioperative period.

**Study selection process**

Titles and abstracts were screened by members of the thematic subgroups assigned to each PICO question. Full text review was performed when eligibility could not be determined from the abstract alone. Disagreements regarding study inclusion were resolved through discussion within the subgroup or consultation with the coordinating committee.

**Risk of bias assessment**

Risk of bias was assessed according to study design using established and validated tools. Randomized controlled trials were evaluated using the Cochrane Risk of Bias tool (RoB 2.0). Observational studies were assessed using the Newcastle-Ottawa Scale or ROBINS I, as appropriate. Systematic reviews were appraised using AMSTAR 2. Risk of bias judgments were performed at the study level and informed the overall certainty of evidence.

**Integration of risk of bias with GRADE**

Risk of bias assessments were incorporated into the GRADE framework together with other domains, including inconsistency, indirectness, imprecision, and publication bias. These domains informed the final certainty rating for each outcome and recommendation. When evidence was limited, heterogeneous, indirect, or imprecise, recommendations were more likely to be classified as conditional, reflecting uncertainty and the need for individualized clinical judgment.

**Consensus process and expert agreement**

The development of recommendations followed a structured consensus process using a modified Delphi methodology. Agreement thresholds and voting procedures are described in the Methods section of the main manuscript. Detailed results of expert voting, including agreement and disagreement rates for each recommendation, are provided in Supplementary Table S1.

**Supplementary Appendix S2**

**Stepwise approach to perioperative hypoxemia**

Intraoperative and early postoperative hypoxemia is common and often multifactorial. A structured stepwise approach facilitates rapid identification of reversible causes and guides timely corrective interventions during anesthesia and perioperative care.

**Step 1. Confirm hypoxemia and signal reliability**

Verify pulse oximetry waveform quality, probe position, peripheral perfusion, and recent changes in FiO_2_ or ventilator settings.

**Step 2. Identify immediate and reversible causes**

Assess airway patency, endotracheal tube position, circuit integrity, presence of secretions, bronchospasm, or recent patient repositioning.

**Step 3. Optimize ventilatory settings**

Reassess tidal volume based on predicted body weight, respiratory rate, and applied PEEP. Consider recruitment maneuvers when appropriate, using the lowest effective pressure and duration, with close hemodynamic monitoring.

**Step 4. Adjust oxygenation strategy**

Titrate FiO_2_ to achieve target oxygen saturation while avoiding unnecessary hyperoxia. Evaluate potential ventilation-perfusion mismatch related to positioning, pneumoperitoneum, or lung collapse.

**Step 5. Evaluate hemodynamic and contextual factors**

Assess cardiac output, intravascular volume status, and the impact of surgical conditions such as pneumoperitoneum, Trendelenburg positioning, or increased intra-abdominal pressure.

**Step 6. Escalation and reassessment**

If hypoxemia persists, reassess the differential diagnosis, involve the surgical team, and consider advanced monitoring or imaging when available.

**Supplementary Appendix S3**

**Management of hypoxemia during one-lung ventilation**

Hypoxemia during one-lung ventilation is frequent and primarily driven by shunt and ventilation-perfusion mismatch. A systematic approach may improve oxygenation while minimizing ventilator-induced lung injury.

**Step 1. Confirm lung isolation**

Verify correct position of the double-lumen tube or bronchial blocker using fiberoptic bronchoscopy.

**Step 2. Optimize ventilation of the dependent lung**

Ensure lung-protective ventilation using tidal volume based on predicted body weight, appropriate respiratory rate, and individualized PEEP titration, avoiding excessive driving pressure.

**Step 3. Manage the non-dependent lung**

When surgically feasible, consider continuous positive airway pressure or oxygen insufflation to the non-dependent lung, balancing oxygenation benefits against interference with the surgical field.

**Step 4. Recruitment and reassessment**

Consider selective recruitment maneuvers of the dependent lung as rescue therapy, with careful monitoring of hemodynamic tolerance and surgical conditions.

**Step 5. Oxygenation targets and FiO_2_**

Adjust FiO_2_ to achieve acceptable oxygen saturation targets while avoiding sustained hyperoxia.

**Step 6. Persistent hypoxemia**

Reevaluate patient positioning, surgical factors, and hemodynamics. Temporary return to two-lung ventilation may be required in refractory cases.
